# Leaping through Tree Space: Continuous Phylogenetic Inference for Rooted and Unrooted Trees

**DOI:** 10.1093/gbe/evad213

**Published:** 2023-12-12

**Authors:** Matthew J Penn, Neil Scheidwasser, Joseph Penn, Christl A Donnelly, David A Duchêne, Samir Bhatt

**Affiliations:** Department of Statistics, University of Oxford, Oxford, United Kingdom; Section of Epidemiology, University of Copenhagen, Copenhagen, Denmark; Department of Physics, University of Oxford, Oxford, United Kingdom; Department of Statistics, University of Oxford, Oxford, United Kingdom; Pandemic Sciences Institute, University of Oxford, Oxford, United Kingdom; Department of Infectious Disease Epidemiology, MRC Centre for Global Infectious Disease Analysis, School of Public Health, Faculty of Medicine, Imperial College London, London, United Kingdom; Center for Evolutionary Hologenomics, Globe Institute, University of Copenhagen, Copenhagen, Denmark; Section of Epidemiology, University of Copenhagen, Copenhagen, Denmark; Department of Infectious Disease Epidemiology, MRC Centre for Global Infectious Disease Analysis, School of Public Health, Faculty of Medicine, Imperial College London, London, United Kingdom

**Keywords:** phylogenetic inference, balanced minimum evolution, gradient descent, distance matrix

## Abstract

Phylogenetics is now fundamental in life sciences, providing insights into the earliest branches of life and the origins and spread of epidemics. However, finding suitable phylogenies from the vast space of possible trees remains challenging. To address this problem, for the first time, we perform both tree exploration and inference in a continuous space where the computation of gradients is possible. This continuous relaxation allows for major leaps across tree space in both rooted and unrooted trees, and is less susceptible to convergence to local minima. Our approach outperforms the current best methods for inference on unrooted trees and, in simulation, accurately infers the tree and root in ultrametric cases. The approach is effective in cases of empirical data with negligible amounts of data, which we demonstrate on the phylogeny of jawed vertebrates. Indeed, only a few genes with an ultrametric signal were generally sufficient for resolving the major lineages of vertebrates. Optimization is possible via automatic differentiation and our method presents an effective way forward for exploring the most difficult, data-deficient phylogenetic questions.

**Significance**

Phylogenetics is vital in life sciences, revealing early life origins and epidemic dynamics. A central challenge in inferring the best tree from a set of data is that exploring the vastness of tree space is computationally hard. Here we recast the exploration and inference problem from a discrete to a continuous space. Our method performs well on unrooted trees and can accurately infer both the tree and the root in ultrametric cases, but at a substantially increased computational cost. Our approach represents a shift in the methodology to explore tree space and opens the possibilities of new efficient forms of inference.

## Introduction

Phylogenetic inference, the task of reconstructing the evolutionary relationships across taxonomic units given observational data, has a wide range of theoretical and practical applications in biology, such as evolution (Cavalli-Sforza and Edwards 1967; Felsenstein 2004; O’Meara 2012), conservation (Rolland et al. 2011), and epidemiology (Grenfell et al. 2004; Faria et al. 2021), and also in comparative linguistics (Mace and Holden 2005) and cultural anthropology (Collard et al. 2006; Morrison 2014). In particular, the COVID-19 pandemic has catalyzed the development of efficient phylogenetic tools and methods to better understand the virus’ origin, spread, and evolution (Lemoine et al. 2021; O’Toole et al. 2021; Attwood et al. 2022; Sanderson 2022; Turakhia et al. 2022; Voznica et al. 2022; De Maio et al. 2023). For biological problems, tree inference is primarily informed by molecular sequence data (i.e., nucleotide or amino acid sequences), for which an extensive body of literature exists (Sanderson and Shaffer 2002; Yang 2006; Yang and Rannala 2012). Other types of biological data such as morphology (Lee and Palci 2015), fossils (Morlon et al. 2011), and auditory communication in animals (Arato and Fitch 2021) can also be used as input.

Two key parameters considered when inferring a phylogenetic tree include the *topology*, the branching pattern that specifies the evolutionary relationships between operational taxonomic units, and *branch lengths*, the amount of evolutionary divergence that occurred between the branching events. A substantial amount of research has been conducted on how to parameterize branch lengths (Bromham and Penny 2003; Dos Reis et al. 2016), especially through the use of various molecular clocks (Zuckerkandl 1962). Similarly, although to a lesser degree, progress has been made on methods for efficient exploration of the space of tree topologies (Stamatakis 2014), which is fundamentally challenging due to its combinatorial complexity. Indeed, for *n* taxa, there are (2n−3)!! possible rooted tree arrangements, where n!! denotes the semifactorial of *n*—even a small dataset of ten taxa can be enumerated by 34 million unique rooted trees. Moreover, finding the global optimal tree is nondeterministic polynomial time (NP)-hard for all major optimality criteria (e.g., maximum parsimony [Foulds and Graham 1982], minimum evolution (ME) [Day et al. 1986], maximum likelihood [Roch 2006]). Methods such as linear programing (Catanzaro et al. 2012) or branch and bound (Hendy and Penny 1982) can provide exact solutions, but are practically limited to problems with ≍15 or fewer taxa. To overcome these challenges, the overwhelming majority of state-of-the-art software (e.g., MrBayes [Huelsenbeck and Ronquist 2001], PAUP [Wilgenbusch and Swofford 2003], BEAST [Drummond and Rambaut 2007], PAML [Yang 2007], RAxML(-NG) [Stamatakis 2014; Kozlov et al. 2019], FastME [Lefort et al. 2015], IQ-TREE [Nguyen et al. 2015; Minh et al. 2020]) rely on hand-engineered search heuristics to perform tree topology optimization or Bayesian analysis. These are traditionally based on subtree pruning and regrafting (SPR) and tree bisection and reconnection (TBR) operations, which have empirically been shown to be the best available methods for exploring tree topology space (Park et al. 2010; Stamatakis 2014).

However, such methods still have limitations. First, hill climbing using heuristic approaches necessitates multiple evaluations of the objective function to pick the best move. While these heuristics are still polynomial, exhaustive exploration of single SPR operations is quadratic in complexity, and paired operations (two sequential SPR changes) are quartic. Second, all the aforementioned tree arrangements are prone to being trapped in local optima and even if a global optimum is found, terraces of trees with identical quality exist (Sanderson et al. 2011). The challenge of exploring tree space is exacerbated when concatenating multiple genes in supermatrices (Rokas et al. 2003; de Queiroz and Gatesy 2007; Chernomor et al. 2016) or when using genomic-scale datasets which require extensive computational resources.

To address these shortcomings, we propose **GradME**, a new direction for tree topology inference which expands the problem space using a continuous rather than discrete parameterization of a phylogenetic tree. Generally, aside from considering metrics (e.g., distances in tree space) (Billera et al. 2001; Chernomor et al. 2016; Dinh et al. 2017; St. John 2017), performing topological search in a continuous tree space has rarely been explored (for recent work in hyperbolic spaces, see Matsumoto et al. 2021; Wilson 2021; Macaulay et al. 2023; Mimori and Hamada 2023). Furthermore, very few approaches have made use of gradient-based tree proposals (Dinh et al. 2017; Zhang and Matsen 2018; Matsumoto et al. 2021; Nesterenko et al. 2022). Although maximum likelihood and Bayesian inference criteria are more popular and generally considered state-of-the-art (Huelsenbeck and Ronquist 2001; Wilgenbusch and Swofford 2003; Drummond and Rambaut 2007; Ayres et al. 2012; Stamatakis 2014; Minh et al. 2020), the GradME framework optimizes tree topology under a balanced minimum evolution (BME) criterion (Pauplin 2000; Desper and Gascuel 2002) using distance matrices as an input. This criterion is well principled (Kidd and Sgaramella-Zonta 1971) but generally performs worse than likelihood-based (Price et al. 2009; Yang and Rannala 2012; Lefort et al. 2015). However, the framing of the ME criterion (Kidd and Sgaramella-Zonta 1971; Rzhetsky and Nei 1992) has been proven to be statistically consistent (Desper and Gascuel 2004; Felsenstein 2004) and has repeatedly shown good (although not state-of-the-art) performance in various settings (Kuhner and Felsenstein 1994; Kumar and Gadagkar 2000; Gascuel and Steel 2006; Lefort et al. 2015).

To better explore the space of possible trees, we expand the space over which we need to search. Our novel vector representation of a phylogenetic tree, Phylo2Vec (Penn et al. 2023), has a natural continuous extension, allowing us to improve the ability to search parts of this space. Appealing to a common analogy that casts the optimal tree search problem as finding a needle in a haystack, our approach observes a much bigger haystack, but the hay is in very large bundles, many of whom have a needle, and for these bundles we have access to a (weak) magnet. Providing details to this analogy, the size of the usual phylogenetic haystack with *n* taxa is (2n−3)!! (Felsenstein 1978b), while we search a much larger haystack of size $(n!{)}^{2}$. There are n! bundles in this larger haystack, each of which contains n! trees, but for any tree, $2^{n-1}$ bundles will contain that tree. Although the proportion of bundles containing a needle shrinks exponentially, we propose a novel approach (Queue Shuffle) that chooses bundles that should be closer to one with a needle. For any given bundle, we also introduce a continuous objective function that can be efficiently traversed using gradient descent approaches (the weak magnet) developed for large-scale machine learning problems (Kingma and Ba 2015; Shazeer and Stern 2018; Loshchilov and Hutter 2019). This continuous objective facilitates enormous changes to tree topology in a single step in a direction that improves the objective function. After searching any given bundle using the continuous objective, we use Queue Shuffle, which improves the switch toward the next bundle to search. This counter-intuitive approach offers a new addition to the existing heuristic methods used for topological inference, outperforming the current state-of-the-art but as currently stands with a larger overall complexity comparable to other ME approaches such as neighbor joining (NJ).

## Results

### Tree Traversal in Continuous Space

**Fig. 1.**
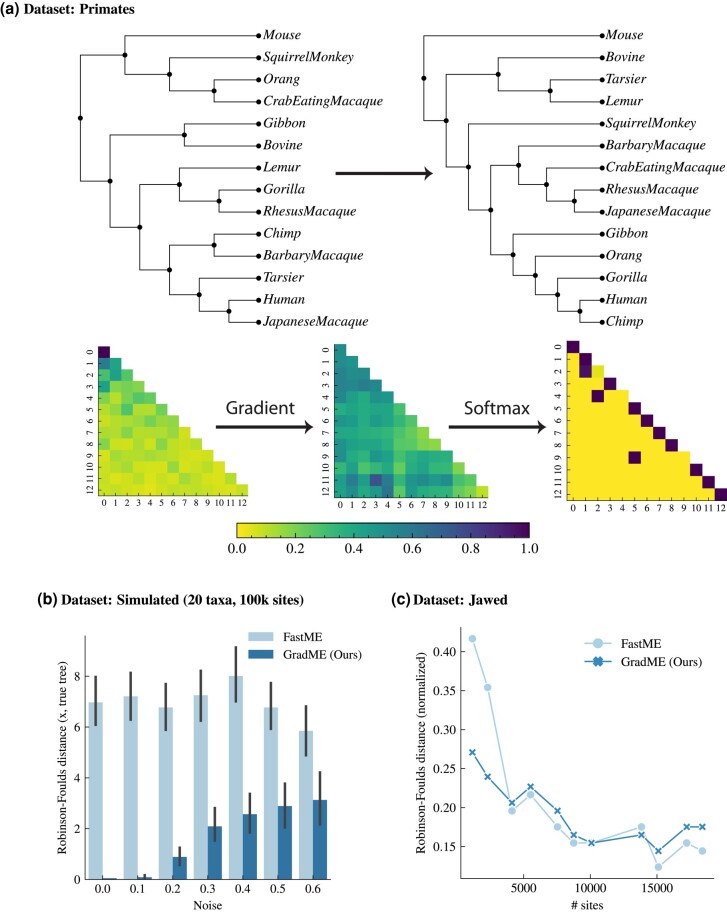
Results on empirical data. (*a*) Starting from a random tree, represented by an n×n stochastic matrix, we compute the continuous gradient, apply softmax activation, and increment the original matrix. In a single step, our gradient finds the correct tree at a distance of six subtree-prune and regraft moves from the random starting tree. (*b*) Simulating ultrametric trees of 20 taxa and 100,000 sites under an Le and Gascuel (LG) model of protein evolution. We add random uniform noise to all branch lengths to simulate departures from ultrametricity. Compared to the true tree via Robinson–Foulds distance, light-coloured bars are midpoint rooting the best FastME tree and dark-coloured bars are the inferred root from our approach. (*c*) Phylogenies for jawed vertebrates, where the number of genes (hence sites) are reduced to be more clocklike. Normalized Robinson–Foulds distance are shown between the best ASTRAL (Zhang et al. 2018) tree, the best unrooted FastME tree which has been midpoint rooted (light colour), and our inferred rooting algorithm (dark colour). Performance for FastME reduces when the number of sites is small.

For any choice of label ordering, our approach admits a continuous gradient across n! trees for *n* leaves. This gradient, which can be obtained readily via automatic differentiation, can rapidly traverse tree space to find trees with a close to optimal objective value. Figure 1*a* shows a single gradient step for the small Primates dataset (Paradis et al. 2004; Yang 2007). Simply subtracting the gradient from a random initial tree, followed by softmax activation, results in an almost discrete *W* which corresponds to the best BME tree for a given substitution model. Note that if more gradient steps were taken, the *W* matrix would quickly become discrete (from supplementary Lemma 5, Supplementary Material online). The jump taken corresponds to six subtree-prune and regraft moves (Paradis et al. 2004).

For larger alignments such as the popular Eutherian dataset (Song et al. 2012), a single gradient step can result in 14–18 SPR moves. While the number of SPR moves achieved is large, this is achieved with a substantial increase in overall computational complexity when compared to FastME. We note that the gradient step size is dependent on the data and, as expected, greatly reduces as we approach an optimum.

### A Comparison to Benchmark Phylogenetic Data Sets

Table 1 presents a comparison of GradME with neighbor joining (BioNJ) and FastME (subtree-prune and regraft version) over 11 popular phylogenetic benchmark datasets (Whidden and Matsen 2015). Both NJ and FastME are only able to infer a minimum length unrooted tree, and therefore we compare estimates only on unrooted trees. We always initialize our algorithm with a uniform, equiprobable tree, where the starting taxon labeling is random and optimized using Queue Shuffle. We estimate tree using distances from a GTR+*Γ* model estimated via maximum likelihood (see supplementary Appendix I, Supplementary Material online for details). As expected, FastME consistently outperforms BioNJ, with lower BME loss on all alignments. On the other hand, GradME always achieves a better or equal loss compared to FastME. We observe similar results when using different substitution models (e.g., F81). In the two examples where GradME does better than FastME, the topological accuracy, measured by one minus the Robinson–Foulds distance (Robinson and Foulds 1981), is close to 0.9, suggesting FastME has converged to a similar tree. We note that FastME’s performance is generally worse when using the nearest neighbor interchange (NNI) heuristic (instead of the SPR-based heuristic). When compared to a maximum likelihood gold standard (IQ-TREE [Minh et al. 2020]), the best distance method does not recover the same tree as that from maximum likelihood, but in some cases, is very close (e.g., DS3 and DS7). Finally, we note that while GradME outperforms FastME, it is orders of magnitude slower and in most of the data sets, FastME finds the same optimal tree as GradME.

**Table 1:** BME loss scores for 11 Phylogenetic Benchmark Datasets

Dataset	BioNJ	FastME	GradME	Topological Accuracy between GradME and FastME	Topological Accuracy between IQ-TREE and the Best Distance Tree
DS1	0.3118613	**0.3101232**	**0.3101232**	1.00	0.54
DS2	3.725205	**3.7239944**	**3.7239944**	1.00	0.77
DS3	8.0115913	**8.0075588**	**8.0075588**	1.00	0.97
DS4	2.2528503	**2.2447615**	**2.2447615**	1.00	0.68
DS5	6.3077156	**6.2606057**	**6.2606057**	1.00	0.70
DS6	0.6249236	0.6228563	**0.6219367**	0.87	0.67
DS7	9.9174641	**9.882138**	**9.882138**	1.00	0.91
DS8	1.337924	**1.3252984**	**1.3252984**	1.00	0.82
DS9	0.3788481	**0.3788481**	**0.3788481**	1.00	0.66
DS10	1.1286037	**1.1247627**	**1.1247627**	1.00	0.78
DS11	1.313921	1.3096422	**1.3096415**	0.88	0.53

Note.—Lower is better. Scores from BioNJ and FastME were obtained following the implementations in ape (Paradis et al. 2004) using the same distance matrix as GradME. The distance matrix was estimated from a GTR+*Γ* model via maximum likelihood (Yang 2006). Our GradME approach always starts from a uniform tree distribution (every tree is equiprobable) with a random taxon ordering (optimized by Queue Shuffle). The best performing approaches for each dataset are denoted in bold. GradME either equaled or performed better than FastME. The topological accuracy, measured as one minus the Robinsons–Foulds distance, is shown between GradME and FastME and GradME and a maximum likelihood gold standard from IQ-TREE also using a GTR+*Γ* model.

### Rooting Ultrametric Trees

Despite being applicable to the unrooted problem, our approach, at its core, works with rooted trees. As previously discussed, if we assume the existence of a distant outgroup, then the BME objective can be used to optimize a rooted phylogenetic tree. In supplementary Appendix A, Supplementary Material online, we show that, given an ultrametric unrooted tree, the optimal rooting maximizes a heuristic for the root-to-tip distance in the tree. Equivalently, the optimal rooting ensures that the root is estimated to be the maximal possible distance back in time. This is not an immediately biologically plausible objective for the root. Indeed, the cornerstone of BME is finding the tree of minimum length, and it hence seems counter-intuitive to require the root that is the maximum distance backwards in time (though this does in fact minimize the tree length). However, our assumption of a distant ancestor means that the root of our tree must be the point that is furthest backwards in time. In particular, this means that the evolutionary direction needs to be away from the root. By setting our root such that the root-to-tip distance is maximized, we ensure that the root satisfies this constraint. However, this property does not hold for trees that are not ultrametric—in these cases, the root will be drawn toward branches with higher mutation rates.

While this property only holds for ultrametric trees, our approach still works well for near clock-like trees. As an experiment, we draw small (20 taxa) random ultrametric phylogenies with a total length of one, and simulate 100,000-residue protein sequences (Paradis et al. 2004) down these trees under an LG (Lee and Palci 2015) model of protein evolution, assuming random uniform amino acid base frequencies. In the ultrametric cases, all taxa are equidistant to the root, which corresponds to a strict molecular clock. We add uniform noise to all branch lengths to simulate departure from a strict clock. Figure 1*b* shows the Robinson–Foulds (Robinson and Foulds 1981) distance from the true tree to the *midpoint rooted* best *unrooted*  FastME tree (when SPR moves were used by FastME), and the distance to our inferred rooted tree. We see that when the tree is ultrametric, or close to ultrametric, our approach recovers the correct rooted tree. As expected, an increase in noise leads to a decrease in topological accuracy, although our approach still performs substantially better than midpoint rooting. We note that uniform noise is unlikely to be biologically realistic. Instead, deviations from a strict clock are more likely to be heterogeneous in certain clades or internal branches. However, for small departures, we believe our algorithm to reliably infer the correct tree and root simultaneously.

We implement our rooting algorithm on the popular mammal data from Song et al. (2012). We infer a rooted tree via Queue Shuffle and also midpoint root the best FastME tree. Both trees, unrooted, have the same BME loss, but our rooted loss is less than the FastME midpoint rooted loss. Our rooted tree correctly identifies *Gallus gallus* (red junglefowl) as the outgroup, while midpoint rooting pairs *Gallus gallus* with *Ornithorhynchus Anatinus* (platypus) (see supplementary fig. S2, Supplementary Material online for the rooted phylogenies).

### Rooting the Phylogeny of all Jawed Vertebrates

To perform a more detailed evaluation of our framework, we tested GradME’s robustness for topological inference by finding the root of the large jawed vertebrates dataset from Irisarri et al. (2017) with 99 taxa and 4593 genes. Given the reliance of our method on ultrametric data for inference of the root, we first made a fast measure of the ultrametricity of each gene-tree. To do this, we inferred the phylogeny of each gene using GradME, followed by midpoint rooting. The coefficient of variation in root-to-tip lengths was taken as a measure of ultrametricity. We then concatenated ranked genes into supermatrices including decreasing numbers of genes, and we examined the performance of GradME with midpoint rooting against our method. All inferences were performed using the LG amino acid substitution model to maintain simplicity. We placed special focus on our ability to use small portions of data for recovering the main groupings of vertebrates; these key groupings include the root separating cartilaginous (Chondrichthyes) versus boned vertebrates, ray-finned fishes (Actinopterygii), and the major groups of tetrapods and amniotes (amphibians, mammals, archosaurs, turtles, and lizards and relatives).

**Fig. 2.**
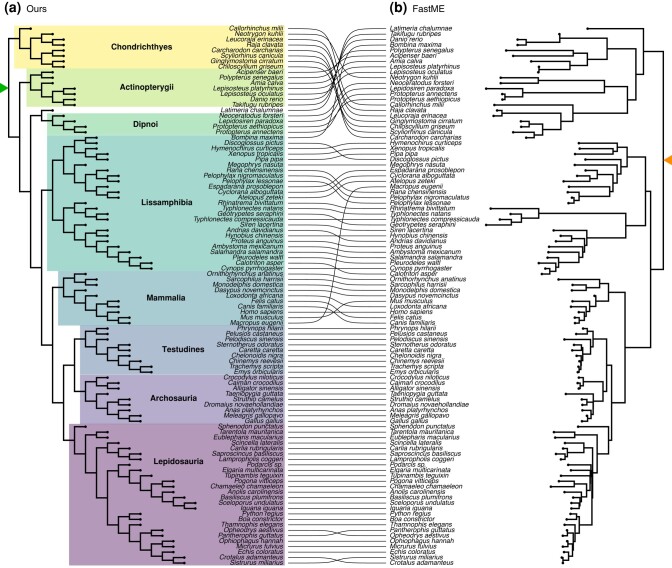
Phylogenetic inferences of the jawed vertebrates’ phylogeny using the two most ultrametric loci from a data set of 99 taxa and 4593 genes (Irisarri et al. 2017). (*a*) Inference using our approach leads to high accuracy in identifying the root and all major jawed vertebrate taxa. Note that we do not estimate branch lengths, but only topology via BME. (*b*) Inference using FastME and midpoint rooting leads to widespread error, primarily and critically near the root of the process.

A small number of genes with an ultrametric signal were generally sufficient for resolving many of the major lineages of vertebrates using both midpoint rooting and our approach (fig. 1*c*). For larger numbers of genes, midpoint rooting and our approach are broadly similar. However, at the smallest numbers of genes (0.05%, two genes), midpoint rooting was unable to recover many of the early relationships among vertebrates, such as the root, monophyly of cartilaginous fishes, ray-finned fishes, Tetrapoda, or the mammals. Even small amounts of data (1460 amino acids of 1,964,439; 0.07% of the original data) were sufficient for GradME to resolve the root as well as every major grouping of jawed vertebrates accurately (fig. 2). The only exception was the controversial position of the Coelacanth, which was found to be sister of Dipnoi (lungfish) rather than the more widely accepted position as sister of Dipnoi plus Tetrapoda. While this remarkable performance under the simple LG model is in part attributed to the informative nature of highly ultrametric genes, our tree topology demonstrates the superiority of our approach in accuracy and efficiency over other fast methods in phylogenetics.

## Discussion

We have introduced a new approach for exploring the vastness of tree space. Counter-intuitively, our approach explores a much bigger space than the space of possible trees, but this larger space allows for new ways to find the best tree. The key to our method’s success lies in transforming the phylogenetic tree search problem from a discrete to a continuous one, allowing us to achieve superior performance. To our knowledge, this is the first time a continuous, differentiable objective function for the inference of tree topology has been proposed, and it opens new possibilities for phylogenetic inference. Bayesian phylogenetics can be regarded as the most robust framework for inferring phylogenies, but has been to-date limited by the poor ability of random walk Metropolis–Hastings algorithms to explore tree space (Betancourt 2018). More efficient Hamiltonian Monte Carlo samplers have been proposed (Dinh et al. 2017) to tackle this problem, and our framework presents a new avenue to jointly explore topology and branch lengths with efficient samplers. A remaining limitation of our approach is the need to shuffle labels to fully explore the space of all possible trees, and while the approach we use, Queue Shuffle, is mathematically and practically powerful, this step is still discrete. The possibility of permutation distributions such as the Gumbel–Sinkhorn distribution could allow for a fully differentiable algorithm. Finally, the complexity of our approach is $O(n^{5})$, which easily allows for large phylogenies up to a thousand, but not tens of thousands. However, computation on graphical processing units (GPUs) in parallel can facilitate computational tractability.

A major benefit of our approach is that it naturally enables the estimation of the root node, which has been a long matter of interest in the biological sciences (Huelsenbeck et al. 2002; Tria et al. 2017; Naser-Khdour et al. 2022). For genes where a strict clock is a reasonable assumption, our method of traversing tree space in large steps reliably estimates both the correct tree topology and the root. Our approach will likely be useful in settings where genetic sequences are contemporaneous and time for measurable evolution is short, such as early epidemics or nosocomial settings. However, as we showed analytically, our approach will have reduced performance when considering rate heterogeneity and departures from a strict clock.

Tests on the relationships among jawed vertebrates demonstrate that even minimal amounts of data can be sufficient for our method to reach high accuracy in topology and root estimates. These results are consistent with previous work on large amounts of genome-scale data showing that clocklike loci to be the most suitable for phylogenetic inference (Vankan et al. 2022). Furthermore, our approach is effective with negligible amounts of data—where other methods are ineffective—making it a powerful addition to the existing toolkit for addressing recalcitrant questions of the tree of life.

Our approach is based on the ME principle, which has repeatedly shown to produce fast and accurate inference. Nonetheless, an interesting area for further study is to extend the continuous path length formulation to approximations of traditional phylogenetic likelihoods (Felsenstein 1983). This would be particularly beneficial for implementation in Bayesian inference, since tree topology inference is a major obstacle to large hierarchical models (Suchard et al. 2003; Duchêne et al. 2016). Our method is therefore a step toward more efficient sampling of the complex posterior distributions over tree topology.

## Methods

In the following, we describe GradME, a distance-based method for continuous phylogenetic inference of rooted and unrooted trees using gradient descent. The framework can be divided into three components: (i) a continuous tree representation based on Phylo2Vec (Penn et al. 2023), a bijective integer representation of phylogenetic trees; (ii) gradient-based optimization using a continuous version of the BME criterion (Pauplin 2000; Desper and Gascuel 2002), and (iii) Queue Shuffle, a method to shuffle the integer-to-taxon mapping underlying Phylo2Vec for full tree space exploration. The overall approach works for both rooted and unrooted trees.

### Balanced Minimum Evolution

Popular objective functions to infer the optimal tree from phylogenetic data include maximum parsimony (Fitch and Margoliash 1967), maximum likelihood (Felsenstein 1983), and ME (Saitou and Nei 1987a). Maximum likelihood and ME are provably statistically consistent (Desper and Gascuel 2004; Felsenstein 2004), whereas maximum parsimony can be inconsistent under certain conditions (Felsenstein 1978a). For small to moderate sized phylogenies, methods based on maximum likelihood (and Bayesian extensions) are generally considered state-of-the-art (Wilgenbusch and Swofford 2003; Drummond and Rambaut 2007; Stamatakis 2014; Minh et al. 2020). However, approaches based on ME have also shown to yield adequate performance (Kuhner and Felsenstein 1994; Kumar and Gadagkar 2000; Gascuel and Steel 2006; Roch 2006; Lefort et al. 2015). The first introductions of the ME paradigm (Kidd and Sgaramella-Zonta 1971; Rzhetsky and Nei 1992) sought to express evolutionary relationships through dissimilarity. They proved that, given unbiased estimates of the true evolutionary distances, the true phylogeny has an expected length shorter than any other possible phylogeny—thereby establishing the principled ME criterion. Currently, the best performing ME approach is that of BME (Pauplin 2000; Desper and Gascuel 2002), with FastME (Lefort et al. 2015) being a popular software implementation. Its objective function can be written as


(1) $$L(T)=\sum_{i,j}D_{ij}2^{-e_{ij}}$$

where $D_{ij}$ denotes a distance (e.g., based on molecular sequence data) between two taxa *i* and *j* and $e_{ij}$ the number of branches in the path between taxa *i* and *j* (the path length [Semple and Steel 2004]). This objective can be computed in a numerically stable fashion using the log-sum-exp trick (see supplementary Appendix K, Supplementary Material online for an example snippet). A widely used approach to estimate the optimal tree greedily (Desper and Gascuel 2004; Gascuel and Steel 2006) is the NJ method (Saitou and Nei 1987a). When NJ is based on an additive distance measure, it reconstructs a unique tree, but still performs well with near-additive trees (Atteson 1999) and under small perturbations in the data (Mihaescu et al. 2009). However, despite these highly favorable properties, further heuristic optimization on a NJ tree using SPR moves have proven to be even more accurate (Lefort et al. 2015). Once a tree topology is found, quadratic algorithms exist for estimating the branch lengths (Mihaescu and Pachter 2008) as well as efficient approaches for molecular clock dating (To et al. 2016).

### BME for Rooted Trees

Inference using BME is always restricted to unrooted trees (Semple and Steel 2004; Catanzaro et al. 2022) with rooting chosen after inference through heuristics (e.g., midpoint rooting) or via a molecular clock (e.g., for serially sampled data). However, it is often of interest to find the optimal rooted tree for a set of taxa, as this provides extra biological context (e.g., to represent evolutionary paths).

In an unrooted tree, the BME objective function (eq. 1) provides an efficient way of calculating the total length of a tree where the branch lengths are the least squares estimators for approximating each $D_{ij}$ with the distance from nodes *i* to *j* in the tree. However, this result does not hold in a rooted tree, as the addition of a root changes many of the path lengths. To remedy this, we consider adding a “root taxon” to the tree by joining it to the root node as taxon *n*. If the tree is roughly ultrametric, then we expect


(2) $$D_{ni}\approx D^{*}\;\forall i\neq n$$

where $D^{*}$ is the (assumed constant) root-to-taxa distance. Of course, we do not know the sequence of the root but, as we will show, the value of $D^{*}$ is unimportant—it is instead simply important that it is independent of *i*. Adding this root taxon as a leaf node transforms the tree from being rooted to being unrooted, where standard BME can be used. From this assumption, we prove two lemmas to ensure the framework’s validity, showing that the optimal unrooted tree is obtained when the variation in the root-to-taxa distance is sufficiently small (supplementary Lemma 1, Supplementary Material online), and subsequently that, in all cases, the optimal rooting for an unrooted tree solves a biologically plausible optimization problem (supplementary Lemma 2, Supplementary Material online).

First, supplementary Lemma 1, Supplementary Material online, shows that if


(3) $$|D_{ni}-D^{*}|<\delta \;\forall i\neq n$$

then, using $e_{ij}^{u}$ and $e_{ij}^{r}$ to denote path lengths in the unrooted tree (*u*) containing taxon *n* and the rooted tree (*r*) formed by removing taxon *n*:


(4) $$\left|\sum_{i=0}^{n}\sum_{j=0}^{n}D_{ij}2^{-e_{ij}^{u}}-\sum_{i=0}^{n-1}\sum_{j=1}^{n-1}D_{ij}2^{-e_{ij}^{r}}-D^{*}\right|\leq \delta$$

where *δ* denotes a small number. Hence, the difference between the rooted and unrooted objective functions is approximately equal to the constant, $D^{*}$. Thus, for sufficiently small *δ*, by the discreteness of tree space, we can see that, if it is unique, the optimal unrooted tree under the *rooted* objective (using $e_{ij}^{r}$) will be the same as that under the unrooted objective (using $e_{ij}^{u}$), when the root taxon is used as an additional leaf.

Subsequently, supplementary Lemma 2, Supplementary Material online, shows that, for the correct unrooted tree, the BME-optimal rooting maximizes a simple heuristic (defined in supplementary Definition 2, Supplementary Material online) for the root-to-tip distance. Equivalently, the optimal rooting ensures that the root is estimated to be the maximal possible distance back in time.

This is not an immediately biologically plausible objective for the root. Indeed, the cornerstone of BME is that we want the tree of *minimum* length, and it hence seems counter-intuitive to require the root that is the *maximum* distance backwards in time (though, by supplementary Lemma 2, Supplementary Material online, this does create the minimum length tree). However, the root of our tree must be the point that is furthest backwards in time. In particular, this means that the evolutionary direction needs to be away from the root. By setting our root such that the root-to-tip distance is maximized, we ensure that the root satisfies this constraint.

This method shares a similar motivation with midpoint rooting, which also seeks to maximize a heuristic for root-to-tip distance. However, the heuristic used in midpoint rooting uses only the two taxa which are furthest apart, while our rooting method uses distances from all taxa. Thus, we expect our method to be more robust, particularly as large inter-taxa distances are difficult to estimate, meaning that the additional information used in our heuristic should help to reduce errors. This is evidenced in supplementary figure S2, Supplementary Material online, where midpoint rooting leads to incorrect root placement.

Nonetheless, this property will not hold if the tree is not ultrametric. If taxa evolve at different rates at different times throughout the tree, then the root will be drawn toward taxa with high evolutionary rates. Thus, caution must be used when applying our rooted algorithm to such trees, although the unrooted algorithm will still give a correct unrooted tree. In this case, it may be best to find the optimal unrooted tree topology and then solve the rooting problem for this tree, rather than finding the optimal rooted tree, as the former will reduce the skewing effect of the heterogeneity in evolutionary rates. Because of this, the algorithm introduced in this paper has the flexibility to find the optimal unrooted or rooted tree.

### An Ordered Bijection to Tree Space

Previously, we introduced Phylo2Vec (Penn et al. 2023), a novel bijection between the space of phylogenetic trees and a space of integer vectors. In contrast to other bijections such as permutation matchings (Diaconis and Holmes 1998), changes in Phylo2Vec correspond to smooth changes in the tree space, for example, single changes in a Phylo2Vec vector correspond to a limited set of SPR changes. On the other hand, Prüfer codes (Chen and Wang 2000) form a bijection to the space of all *m*-ary trees, meaning that there is no guarantee to sample binary trees from random Prüfer sequences.

**Fig. 3.**
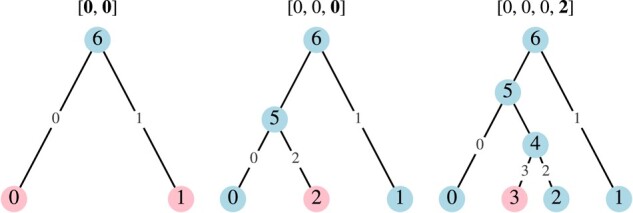
An example of the left-to-right construction of the ordered tree v=[0,0,0,2].

Here, we focus on the notion of *ordered trees* from Penn et al. (2023), where it is possible to construct a tree from its vector in linear time. An ordered tree can be thought of as a birth process, such that when a birth occurs, the original node continues to live and retains its label, while the new node receives an incremented label. Accordingly, we introduce an equivalent but more intuitive tree construction process for these ordered trees (see fig. 3 for an example). We begin with two leaf nodes and two edges labeled 0 and 1. We then append nodes by joining them, *in order*, to edges connecting leaf nodes to the tree. This tree construction process can be summarized by a single vector v, with $v_{0}=v_{1}=0$ and, for m≥2, $v_{m}$ being the label of the edge to which node *m* is appended. Each index in $v_{m}$ is subject to the simple constraint:


(5) $$\begin{array}{cc} & v_{0}=v_{1}=0\\ \text{and} & v_{m}\in \{0,1,\ldots ,m-1\}\;\forall \;m\geq 2 \end{array}$$

which is equivalent to the definition of ordered trees in Penn et al. (2023). Intuitively, a tree is ordered if, starting with a branch connecting the root to taxon 0, the taxa can be added in order of their label by appending a new branch to a *terminal* branch of the existing tree (i.e., a branch connecting a leaf node to the rest of the tree). Thus, the ordering of the taxa is in some sense “natural” for each possible ordered tree. It is proved in supplementary Lemma 3, Supplementary Material online that for ordered v, the algorithm presented above and the more general Phylo2Vec algorithm in Penn et al. (2023) produce equivalent trees.

Note that, for a fixed integer-taxon labeling, the number of ordered trees is a subset of the number of possible trees. We discuss an efficient method to remedy this problem and explore all tree space called the Queue Shuffle.

### A Continuous Representation of a Tree

We introduce a continuous, probabilistic, representation of trees using a square matrix *W* which gives the distribution of a random ordered vector v with independent entries such that $W_{ij}=P(v_{i}=j)$. Given equation (5), *W* is a lower-triangular, stochastic matrix (row sums to 1). Thus, *W* can probabilistically represent any *ordered* phylogenetic tree (a space of (n−1)! trees). A simple approach to determining the most likely single tree from *W* is to take the column-wise argmax, yielding a single tree v.

### Gradient-Based Optimization Using the BME Criterion

Using this continuous representation, we can find the optimal ordered tree. Defining f(v) to be the BME objective function for the tree generated by an ordered vector v, we then create a continuous objective, F(W) by F(W)=E[f(v)].

The calculation of F(W) follows our new method of constructing ordered trees from the vector v. For a fixed (randomly chosen) tree, we define $e_{ij}^{k}$ to be the path length between nodes *i* and *j* when nodes 0,1,…,k−1 have been added to the tree (for i,j<k). Note that to find the rooted objective function, we initialize with $e_{10}^{2}=e_{01}^{2}=2$, while to find the unrooted objective, we initialize with $e_{10}^{2}=e_{01}^{2}=1$ (while the Phylo2Vec representation is an inherently rooted representation, this unrooted objective finds the tree length if the root were removed from the random rooted tree with distribution given by *W*). This is because, in a tree where the only leaf nodes are 0 and 1, these nodes are a path length of 2 apart if there is also a root (as the root is on this path) while otherwise, they are a path length of 1 apart.

If node *k* is appended to the edge joining either node *i* or *j* to the tree, then $e_{ij}^{k+1}=e_{ij}^{k}+1$; otherwise, $e_{ij}^{k+1}=e_{ij}^{k}$. Similarly, if node *k* is appended to the edge joining node *i* to the tree, then $e_{ik}^{k+1}=2$ and otherwise, if node *k* is appended to the edge joining node *x* to the tree, then $e_{ik}^{k+1}=e_{ix}^{k}+1$. Thus, using $V_{k}$ to denote the random value of $v_{k}$, we can write


(6) $$e_{ij}^{k+1}=e_{ij}^{k}+G_{ij}^{k}(V_{k})$$

(7) $$\Rightarrow \;e_{ij}^{n-1}=\sum_{k=2}^{n-2}G_{ij}^{k}(V_{k})+e_{ij}^{2}$$

for some functions $G_{ij}^{k}$ which are derived explicitly in supplementary Lemma 4, Supplementary Material online. Importantly, each term in the sum is independent, and hence, in supplementary Lemma 4, Supplementary Material online, a closed iterative system for the quantities $E_{ij}^{k}=E(2^{-e_{ij}^{k}})$ can be calculated for i<j as


(8) $$E_{ij}^{k+1}=\left\{ \begin{array}{cc} E_{ij}^{k}\left[1-\frac{1}{2}(W_{ki}+W_{kj})\right] & \text{if }i<j<k\\ \left[\frac{1}{2}\sum_{x\neq i}E_{ix}^{k}W_{kx}\right]+\frac{1}{4}W_{ki} & \text{if }i<k \end{array}\right.$$

with the remaining values for i>j following by symmetry.

The objective function is a polynomial function of the entries of *W* and is linear in each fixed entry (that is, the diagonal entries of $\nabla^{2}F$ are zero). Thus, by supplementary Lemma 5, Supplementary Material online, there is always a minimum at a “discrete tree” (that is, at a matrix *W* where for each row, one value is 1 and all the others are 0). Moreover, this simple form makes it easy to differentiate *F* analytically, numerically, or automatically. Using state-of-the-art automatic differentiation (Bradbury et al. 2018), gradient descent can be used to efficiently minimize *F* and find the optimal *ordered* tree.

There may also be minima at non-discrete trees if multiple trees share the same, optimal, objective value. In our rooted optimization, this is highly unlikely (as two topologically different trees having equal objectives places a dimension 1 condition on the distance matrix *D*, meaning the set of distance matrices for which this happens has measure 0 in the set of possible distance matrices), but when we use our unrooted algorithm, this will occur. This is because, as discussed in Penn et al. (2023), there are n−1 Phylo2Vec vectors which, when the root is removed, give the same unrooted tree. Thus, if multiple rooted vectors giving the same unrooted tree U are in the same space of ordered trees, then, if U is the optimal tree under this ordering, the algorithm may converge to a non-discrete *W*. In this case, taking the argmax safely recovers an optimal rooted tree as, by supplementary Lemma 5, Supplementary Material online, all possible trees according to *W* will have the optimal objective value.

The tree space induced by our continuous objective function will have local minima whenever changing any single entry of the vector v causes the objective function to increase. As the Hamming distance between vectors u and v is comparable to SPR distance (Penn et al. 2023), we therefore expect that the discrete subset of our continuous tree space will be similar in structure to the space induced by SPR moves. However, by starting from a uniformly distributed tree, where all possible ordered trees contribute to the objective, we expect that our algorithm is better able to pick up the “signal” from the true optimum, and avoid moving toward suboptimal local minima.

A Python-like algorithm to compute $E(2^{-e})$ is shown in Algorithm 1. To find the $E_{ij}^{m}$ terms, there are O(m) steps of O(m) (finding the $E_{m-1,j}^{m}$ terms) and $O(m^{2})$ steps of O(1) (finding the other $E_{ij}^{m}$ terms). There are O(n) values of *m* that need to be considered, and hence this system can be solved in $O(n^{3})$ time.

**Algorithm 1:** Compute $E:=E(2^{-e})$

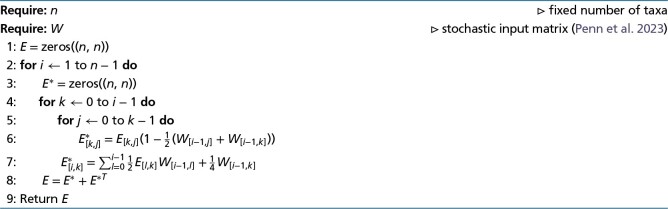

### Orderings

The continuous objective function is only defined for ordered trees, which, for a given labeling of the nodes, is a subset of the whole tree space. Thus, we define the concept of an *ordering* of the nodes, whereby changing the ordering allows the full space of trees to be explored.

Definition 1 **Ordering:** Suppose that the nodes correspond to taxa with names $N_{0},N_{1},\ldots N_{n-1}$. We then define an *ordering* of the nodes to be a permutation, *σ*, of the set {0,…,n−1} such that the node with name $N_{i}$ is processed as node σ(i) by the Phylo2Vec algorithm. It is necessary that the associated Phylo2Vec vector v(σ) is ordered.

Note that we use the phrase “node *x*” to mean “the node with name $N_{x}$” and will always be explicit if we refer to a node by its label.

Given a tree, it is possible to generate a possible ordering of the nodes, *σ*, as well as the associated vector v(σ). We will do this by labeling the tree as follows, again distinguishing between the leaf node names $N_{i}$ and their labels (which we will call l(i)).

Consider labeling the root node as 0. Then, label the children of this node as 0 and 1. One can continue this process inductively, choosing a node, labeled *x*, with unlabeled children and then labeling its children as *x* and *y*, where *y* is the smallest unused label. This process terminates when every node has been labeled. As discussed above, suppose that the label of the leaf node with name $N_{i}$ is l(i). Then, supplementary Lemmas 6–8, Supplementary Material online shows that *l* is a possible ordering of the tree and provide a method for calculating the associated ordered Phylo2Vec vector v(l).

Given a tree, one can use the previous labeling algorithm to show that there are at least $2^{n-1}$ possible orderings for the tree (as the children of each node can be labeled in either order). The exact number depends on the choice of which internal node is processed at each step (and so, a “balanced” tree where the leaves have a low generation has more possible orderings than a “ladder” tree where each leaf has a distinct generation). However, in general, it will be substantially larger than $2^{n-1}$ (we conjecture that for flat trees, it may be factorial in size) and hence there are numerous possibilities which will allow for a global minimum of the objective to be found.

### Queue Shuffle: Changing Orderings to Explore all the Tree Space

The number of ordered trees (different from ranked trees [Collienne and Gavryushkin 2021]), (n−1)!, is substantially smaller than the possible number of trees, (2n−3)!! (albeit with a comparable growth pattern in *n*), and hence, while the optimal ordered tree will be closer to the true tree than a very large proportion of trees, it is very unlikely to be exactly equal to the true tree.

To fully explore tree space, one must shuffle the labels of the leaf nodes in the optimal ordered tree. Simply choosing a uniformly random permutation will lead to extremely inefficient optimization, as each tree is only possible in approximately $1/2^{n}$ of the possible orderings. Instead, we use the topology of the optimal tree to inform our choice of permutation through a novel approach we call the *Queue Shuffle*. This ensures that the previous optimal tree can be written as an ordered tree in the new ordering, while also ensuring a smooth and efficient path through the space of orderings.

The Queue Shuffle is motivated by the labeling procedure discussed in the previous section, but ensures that the set of internal nodes with a given generation (that is, a given distance from the root) are processed consecutively. That is, we begin by processing all nodes with generation 0 (i.e., the root), then all internal nodes with generation 1, then all internal nodes with generation 2, and continue in this fashion until all internal nodes have been processed.

Algorithmically, this can be achieved by a “queue” of internal nodes to be processed. When an internal node is processed, any of its children that are also internal nodes are added to the back of this queue. Thus, the queue is always in ascending order of generation, and it is simple to show that this ensures that nodes are processed in non-decreasing order of generation.

A crucial feature of this queue is that the child given the same label as its parent is placed *ahead* of the other child in the queue. This ensures that one can, in some way, control the order of processing by choosing the labeling of the children of each node. Moreover, it is vital for the theoretical result presented in the following section.

To add randomness into the labeling procedure, every time an internal node is processed, we randomly choose which child is given the label of their parent, and which child is given the next available label. This provides $2^{n-1}$ possible orderings for each tree. This stochasticity is helpful in ensuring that the algorithm does not get stuck—as discussed in the subsequent section, it ensures that a large class of similar trees will be considered after a few ordering proposals.

An algorithmic description of the Queue Shuffle is provided in Algorithm 2.

**Algorithm 2:** The Queue Shuffle

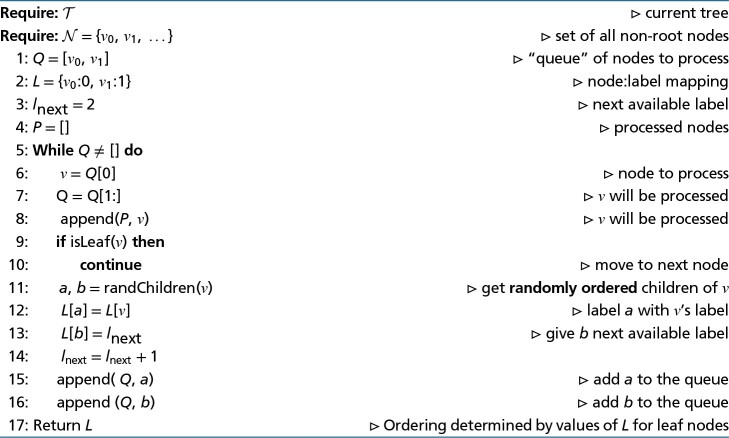

### GradME

The Queue Shuffle completes our optimization algorithm. We iteratively find the best ordered tree according to the current ordering and then use Queue Shuffle to change ordering, changing the space of explorable trees. The algorithm terminates when the optimal tree has not been improved upon for a fixed number of iterations (note that, by construction, the previous optimal tree will always be in the new space of ordered trees). In the examples presented in this paper, only ten iterations are needed from some random starting order, and less if a sensible starting ordering (such as from a NJ tree) is used.

We refer to the resulting system, combining the continuous tree representation, queue shuffle reordering, and the gradient-based optimization framework using BME, as **GradME**.

### Why Does Queue Shuffle Work?

A given tree is in the space of ordered trees for at least $2^{n-1}$ orderings. This means that we do not need to find a single optimal ordering, but have exponentially many which will return the true optimal tree. Very loosely considered, being able to explore n! tree space reliably and efficiently with continuous optimization, Queue Shuffle reduces the inferential task to one that is exponential.

However, while the number of optimal orderings grows exponentially, their proportion tends quickly to zero as *n* grows. It is therefore, perhaps, surprising that we are able to find an optimal ordering so quickly from merely tens of shuffles. The proportion of optimal orderings (approximately the ratio of ordered trees to total trees, (n−1)!/(2n−3)!!) ranges from $8\times 10^{-4}$ in our smallest dataset (14 taxa) to $6\times 10^{-29}$ in the largest (99 taxa; see table 2).

**Table 2:** Evaluation Datasets

Dataset (Reference)	# Sites	# Taxa	Type	Taxonomic Rank
DS1 (Hedges et al. 1990)	1,949	27	rRNA (18S)	Tetrapods
DS2 (Garey et al. 1996)	2,520	29	rRNA (18S)	Acanthocephalans
DS3 (Yang and Yoder 2003)	1,812	36	mtDNA	Mammals; mainly Lemurs
DS4 (Henk et al. 2003)	1,137	41	rDNA (18S)	Fungi; mainly Ascomycota
DS5 (Brower 2000; Lakner et al. 2008)	378	50	DNA	Lepidoptera
DS6 (Zhang and Blackwell 2001)	1,133	50	rDNA (28S)	Fungi; mainly Diaporthales
DS7 (Yoder and Yang 2004)	1,824	59	mtDNA	Mammals; mainly Lemurs
DS8 (Rossman et al. 2001)	1,008	64	rDNA (28S)	Fungi; mainly Hypocreales
DS9 (Ingram and Doyle 2004)	955	67	DNA	Poaecae (grasses)
DS10 (Suh and Blackwell 1999)	1,098	67	DNA	Fungi; mainly Ascomycota
DS11 (Kroken and Taylor 2000)	1,082	71	DNA	Lichen
Eutherian (Song et al. 2012)	1,338,678	37	DNA	Eutherian mammals
Jawed (Irisarri et al. 2017)	1,460–18,406	99	AA	Gnathostomata (jawed vertebrates)
Primates (Hasegawa and Kishino 1989; Paradis et al. 2004)	232	14	mtDNA	Mammals; mainly primates

Note:—rRNA/rDNA, ribosomal RNA/DNA; mtDNA, mitochondrial DNA; AA, amino acid. For the Jawed dataset, several subsets of the original dataset (Irisarri et al. 2017) were used (from 1,460 to 18,406 sites; cf. fig. 1*c*).

This efficiency comes from the topology-dependence of the Queue Shuffle algorithm, which allows us to plot a relatively “smooth” path through the space of possible orderings. That is, the majority of trees in the new ordered space will have similar properties to the previous optimal tree and so, unless the previous tree was a local minimum of the objective, it is likely that one of these “close” trees will have a lower objective value.

Supplementary Lemma 9, Supplementary Material online, shows that the expected distance from the root grows harmonically as the label increases. For large trees, the node with label n−1 has an expected distance from the root of approximately twice the expected distance from the root of the node label 0. This property is noticeable even for small trees—if n=10, then the ratio of the expected distance to the root of node 9 and node 0 is approximately 1.65. Thus, nodes which are close to the root in the current optimum will also be closer on average to the root in the new space of ordered trees. In essence, this means that “fewer slots are wasted” in the new ordered space—that is, there are fewer trees in the new space of ordered trees which are topologically far from the previous optimum (a tree that, after the first few iterations, is likely to be far closer to the true optimum than a randomly chosen tree) and hence, more trees which are reasonable candidates for having lower objective values.

Supplementary figure S1, Supplementary Material online, proves another example of the smoothness in transitions induced by the Queue Shuffle, based on NNI moves. An NNI move considers the four subtrees attached to two non-root nodes that share an edge and swaps two of these subtrees. Supplementary figure S1, Supplementary Material online, shows that, starting from a tree T, any tree which is one NNI move away from T will be in the new space of ordered trees with probability at least 1/4.

This ensures that this new space contains many sensible proposal trees. Perhaps the most surprising aspect of this theorem is that this probability is bounded below, independently of the topology. Thus, with high probability, the optimal tree will only remain the same for more than a few iterations if large sets of similar trees yield lower objective values than the current optimum.

That being said, the Queue Shuffle does not guarantee that the global minimum will be found, even if the gradient-based algorithm for optimizing F(W) always converges to the optimal tree. If a tree is “far” from the nearest tree with a better objective value, then it may take a very large number of shuffles (or, indeed, it may be impossible) to find a better tree. However, while the only theoretical guarantee is that supplementary figure S1, Supplementary Material online shows it will quickly find better trees that can be formed by NNI, we expect that stronger conditions hold on its ability to “escape” from local minima.

### Computational Complexity

The computational complexity for all distance-based algorithms requires an upfront computational cost of $O(n^{2})$ to compute the distance matrix. We will disregard this cost from subsequent comparisons. The standard NJ algorithm (Saitou and Nei 1987b) has an overall computational complexity of $O(n^{3})$. FastME has a computational complexity of $O(kn_{\mathrm{Diam}}^{2}(T))$ (where Diam(T) is the maximum path length in a tree, which is generally much smaller than *n*) for *k* iterations where k<n when *n* is large. When fully discrete, our algorithm also has same complexity but with added mechanisms for escaping optima via Queue Shuffle. Therefore, a discrete setting is as computationally efficient as FastME (see supplementary Appendix I, Supplementary Material online for details).

Computing the expectation in Algorithm 1 has complexity $O(n^{3})$. A single gradient evaluation (that is, calculating $\partial F/\partial W_{ij}$ for some *i* and *j*) is also $O(n^{3})$ and therefore computing the full Jacobian is $O(n^{5})$. Our Queue Shuffle algorithm runs in O(n). Therefore, our optimization for *k* steps and *l* shuffles yields a complexity of $O(kln^{5})$. The size of *l* is dependent on the choice of gradient optimizer, and the size of *k* varies if a sensible ordering is initialized.

Thus, the computational complexity of GradME is substantially higher than that of FastME and closer to that of FITCH (Felsenstein 1997). This is due to the far greater mathematical complexity of the continuous objective function, F(W). As it is an expectation over all possible ordered trees, the explicit formula for F(W) is a polynomial in *W* with (n−1)! different terms. Intuitively, the continuous space always considers a path between any two trees, something that becomes impossible with discrete settings. Thus, being able to compute it in polynomial time is a vast improvement on a naive approach, although it is still considerably less than the innovative FastME greedy approach. More savings should be possible, we hope to make further efficiency gains in future work.

### Evaluation

We evaluate GradME on a diverse corpus of 14 empirical molecular sequence datasets (table 2). The first 11 are commonly used to assess phylogenetic inference performance (Whidden and Matsen 2015), whereas the last three were used to assess inference on rooted trees. For each dataset, we start from a random tree and optimize the *W* matrix to a tolerance of $1\times 10^{-10}$ using gradient descent with Adafactor (Shazeer and Stern 2018) optimization. The distance matrix *D* is computed using the GTR+*Γ* substitution model for DNA and an LG model (Le and Gascuel 2008) for amino acids. Substitution model parameters for the GTR+*Γ* are also estimated using gradient descent with Adafactor using a pairwise maximum likelihood approach (Yang 2006). Jukes-Cantor (Jukes and Cantor 1969), F81 (Felsenstein 1981), and TN93 (Tamura and Nei 1993) models were also tested for DNA, while stochastic gradient descent, RMSprop (Tieleman and Hinton 2012), and AdamW (Kingma and Ba 2015; Loshchilov and Hutter 2019) were also considered for optimization (see supplementary fig. S3, Supplementary Material online). To fairly assess the performance of GradME, we compare our framework to two well-established distance-based methods: BioNJ (Gascuel 1997), based on the NJ algorithm (Saitou and Nei 1987b), and FastME (Lefort et al. 2015), based on BME.

### Implementation

Implementation of the BME criterion and the optimization framework was written in Python using Jax (Bradbury et al. 2018) and Optax (Babuschkin et al. 2020). Optimization was performed on a Xeon 2.30 GHz (CPU; Intel Corporation) or on a single GeForce GTX 1080 (GPU; Nvidia Corporation). Evaluation of the BioNJ (Gascuel 1997) and FastME (Lefort et al. 2015) methods was performed via the R package ape (Paradis et al. 2004) using rpy2 (Gautier and Krassowski 2021). Tree manipulation and visualization scripts were written using ete3 (Huerta-Cepas et al. 2016) and NetworkX (Hagberg et al. 2008). An implementation is available at: https://github.com/Neclow/GradME.

## Supplementary Material

evad213_Supplementary_Data

## Data Availability

All code relevant to reproduce the experiments is available online: https://github.com/Neclow/GradME.
