# Supplementary material for: Leaping through Tree Space: Continuous Phylogenetic Inference for Rooted and Unrooted Trees
Source: Genome Biol Evol. 2023 Dec 12;15(12):evad213. doi: 10.1093/gbe/evad213 (PMC10745275; doi:10.1093/gbe/evad213)
Supplement: evad213_Supplementary_Data [file evad213_supplementary_data.zip › GradME__GBE_appendix.pdf]

Appendix

A. BME for rooted trees

Comparing the rooted and unrooted objectives.

Lemma 1: Consider adding an extra taxon, n, to the set of taxa such that, for some D^* and δ.

$$|D_{ni} - D^*| < \delta \quad \forall i \neq n \tag{9}$$

This creates an unrooted tree $\mathcal{T}^u$, and we can create a rooted tree $\mathcal{T}^r$ by removing node n. If e^u_{ij} denotes inter-taxa distance in $\mathcal{T}^u$ and e^r_{ij} denotes inter-taxa distance in $\mathcal{T}^r$, then

$$\left| \sum_{i=0}^{n} \sum_{j=0}^{n} D_{ij} 2^{-e^u_{ij}} - \sum_{i=0}^{n-1} \sum_{j=0}^{n-1} D_{ij} 2^{-e^r_{ij}} - D^* \right| \leq \delta \tag{10}$$

Proof: Firstly, note that for $i, j < n$,

$$e^r_{ij} = e^u_{ij} \tag{11}$$

as the path between i and j will not contain any leaf nodes other than i and j, and therefore will not contain node n. Then, using the fact that $D_{nn} = 0$,

$$\sum_{i=0}^{n} \sum_{j=0}^{n} D_{ij} 2^{-e^u_{ij}} = \sum_{j=0}^{n-1} D_{nj} 2^{-e^u_{nj}} + \sum_{i=0}^{n-1} D_{in} 2^{-e^u_{in}} + \sum_{i=0}^{n-1} \sum_{j=0}^{n-1} D_{ij} 2^{-e^u_{ij}} \tag{12}$$

$$\leq \sum_{j=0}^{n-1} (D^* + \delta) 2^{-e_{nj}} + \sum_{i=0}^{n-1} (D^* + \delta) 2^{-e^u_{in}} + \sum_{i=0}^{n-1} \sum_{j=0}^{n-1} D_{ij} 2^{-e^r_{ij}} \tag{13}$$

We can make progress with this sum by noting the Kraft Equality, found throughout the literature (Catanzaro, Labbé, et al. 2012)

$$\sum_j 2^{-e^u_{nj}} = \frac{1}{2} \tag{14}$$

This means that

$$\sum_{i=0}^{n} \sum_{j=0}^{n} D_{ij} 2^{-e^u_{ij}} \leq \sum_{i=0}^{n-1} \sum_{j=0}^{n-1} D_{ij} 2^{-e^r_{ij}} + D^* + \delta \tag{15}$$

Similarly, one can show

$$\sum_{i=0}^{n} \sum_{j=0}^{n} D_{ij} 2^{-e^u_{ij}} \geq \sum_{i=0}^{n-1} \sum_{j=0}^{n-1} D_{ij} 2^{-e^r_{ij}} + D^* - \delta \tag{16}$$

and hence the result follows.

Understanding the BME rooting.

Definition 2: **Distance to root heuristic:** Under the assumption that the tree is ultrametric, we estimate the distance between the root and the leaf taxa using the following algorithm:

1) Find two leaf nodes that share a parent that is not the root. If no such pair exists, then move to step 3.

2) Let d_1 and d_2 be the distance between each leaf node and its parent. Suppose that d_3 is the distance between the parent and its parent (i.e. the grandparent of the leaves). Remove the leaves from the tree, update the distance between the parent and its parent to be $d_3 + \frac{d_1+d_2}{2}$ and return to step 1

3) If d_1 and d_2 are the distances between the two remaining children and the root, then the distance between the root and the taxa is $\frac{d_1+d_2}{2}$.

Lemma 2: Consider the true unrooted tree $\mathcal{T}$ and suppose that the distance matrix D gives the true (time) distances between each pair of taxa in $\mathcal{T}$.

Then, the optimal rooting — that is, the edge in the tree such that placing the root on this edge minimizes the BME objective — minimizes the distance to root heuristic defined in Definition 2.

Proof: Choose an edge, b on which to place the root. We define an indicator function X_b such that $X_b(A, B)$ is 1 if b is on the path between nodes A and B and 0 otherwise. Adding a root to edge b changes the objective function by halving the weight assigned to each D_{AB} such that $X_b(A, B) = 1$ as the path length between these nodes will increase by 1. Thus, using f_r to denote the rooted objective and f_u to denote the unrooted objective,

$$f_r = f_u - \frac{1}{2} \sum_{X_b(A,B)=1} D_{AB} 2^{-e_{AB}} \quad \textbf{(17)}$$

where the e_{AB} are the path lengths in the original, unrooted tree and both A and B are allowed to vary in the sum. Hence, for a fixed unrooted tree topology, the optimal rooting will solve

$$\max_b \left\{ \sum_{X_b(A,B)=1} D_{AB} 2^{-e_{AB}} \right\} \quad \textbf{(18)}$$

By Lemma 11,

$$\sum_{X_b(A,B)=1} D_{AB} 2^{-e_{AB}} = 2 \sum_A 2^{-g_A} D_{Ar} \quad \textbf{(19)}$$

while, by Lemma 12, the root-to-tip heuristic, $\mathcal{D}$, satisfies

$$\mathcal{D} = \sum_A 2^{-g_A} D_{Ar} \quad \textbf{(20)}$$

and hence, the optimal rooting solves

$$\max_b \left\{ \mathcal{D} \right\} \quad \textbf{(21)}$$

as required.

B. Ordered Trees

Definition 3: **Left-to-right construction algorithm** An ordered tree can be constructed as follows:

1) Begin with nodes 0 and 1, each joined to a root. Label the edge joining node 0 to the root as edge 0, and the edge joining node 1 to the root as edge 1

2) Process the nodes in order 2, 3, 4... When processing node k, join it to edge v_k (creating a new internal node — this is well-defined as $v_k < k$). Label the edge joining node k to the tree as edge k.

Lemma 3: The left-to-right algorithm in Definition 3 and the standard Phylo2Vec algorithm defined in (Penn et al. 2023) produce the same tree, provided $\boldsymbol{v}$ is ordered.

Proof: To show that the left-to-right algorithm gives an equivalent tree, define $\mathcal{T}$ to be the tree resulting from the standard Phylo2Vec algorithm and $\mathcal{T}'$ to be the tree resulting from this new left-to-right algorithm. We proceed by induction on the number of nodes, n, noting that the case $n = 2$ is trivial.

Suppose now that the algorithms are equivalent for $n = m$. Choose some ordered $\boldsymbol{v}$ of length $m + 1$ (so that this corresponds to $n = m + 1$) and consider processing the first node using the Phylo2Vec algorithm, so that (using the fact that $\boldsymbol{v}$ is ordered so that no nodes are skipped), node m merges with node v_m.

From this step, the Phylo2Vec algorithm proceeds as if there were $n-1$ nodes and the vector was $\tilde{\boldsymbol{v}} = (v_0, v_1, ..., v_{m-1})$. Define $\tilde{\mathcal{T}}$ to be the tree given by $\tilde{\boldsymbol{v}}$. Hence, $\tilde{\mathcal{T}}$ can be created from $\mathcal{T}$ by removing nodes v_m and m (and the edges connecting them to the tree) and relabelling their parent as v_m. Equivalently (by reversing this process), $\mathcal{T}$ can be created from $\tilde{\mathcal{T}}$ by adding a node to the edge joining leaf node v_m to the tree, and connecting node m to this edge.

Moreover, from the inductive hypothesis, $\tilde{\mathcal{T}}$ can be constructed by using the left-to-right algorithm on $\tilde{\boldsymbol{v}}$. As this algorithm processes v_m last, this means that after m nodes have been added to the tree by the left-to-right algorithm applied to $\boldsymbol{v}$, the current tree is given by $\tilde{\mathcal{T}}$.

The final step of the left-to-right algorithm applied to $\boldsymbol{v}$ is to add a node to the edge joining leaf node v_m to the tree, and to connect node m to this edge. As previously discussed, this creates the tree $\mathcal{T}$ and hence, $\mathcal{T} = \mathcal{T}'$ as required.

C. A continuous objective function

Construction.

Lemma 4: For a randomly chosen tree with distribution W, define, for $i, j, < k$, e^k_{ij} to be the path length between taxa i

and j when k nodes have been added to the tree (using the left-to-right construction algorithm). Define $E_{ik}^k := \mathbb{E}(2^{-e_{ij}^k})$. Then, for $i < j$

$$E_{ij}^{k+1} = \begin{cases} E_{ij}^k \left[1 - \frac{1}{2}(W_{ki} + W_{kj})\right] & \text{if } i < j < k \\ \left[\frac{1}{2}\sum_{x \neq i} E_{ix}^k W_{kx}\right] + \frac{1}{4} W_{ki} & \text{if } i < k \end{cases} \tag{22}$$

with the remaining values following by symmetry.

Proof: Adding node k to the tree increases the path length between nodes i and j by 1 if and only if $V_k = i$ or $V_k = j$. As this condition is independent of other values of $\boldsymbol{V}$, using e_{ij}^k to be the path length after the k nodes $\{0, ..., k-1\}$ have been added, one can write

$$2^{-e_{ij}^{k+1}} = 2^{-(e_{ij}^k + \mathbb{I}\{V_k \in \{i,j\}\})} = 2^{-e_{ij}^k} \times 2^{-\mathbb{I}\{V_k \in \{i,j\}\}} \tag{23}$$

This is a product of independent random variables and so, defining $E_{ij}^k := \mathbb{E}(2^{-e_{ij}^k})$ and noting that $\mathbb{I}\{V_k \in \{i,j\}\}$ is a Bernoulli random variable with probability $W_{ki} + W_{kj}$, this equation becomes

$$E_{ij}^{k+1} = E_{ij}^k \left[(1 - (W_{ki} + W_{kj}) + \frac{1}{2}(W_{ki} + W_{kj})\right] = E_{ij}^k \left[1 - \frac{1}{2}(W_{ki} + W_{kj})\right] \tag{24}$$

To close this iterative system, note that when node j is added to the tree, it will be a path length 2 from the leaf node V_j connecting the edge it is joined to. Moreover, the distance between node j and any other nodes $x \neq V_j$ in the tree will be equal to one plus the distance between node x and node V_j. That is,

$$2^{-e_{ij}^{j+1}} = \begin{cases} 2^{-(1+e_{i,V_j}^j)} & \text{if } V_j \neq i \\ \frac{1}{4} & \text{if } V_j = i \end{cases} \tag{25}$$

Thus, conditioning on the value of V_j,

$$E_{ij}^{j+1} = \left[\frac{1}{2}\sum_{x \neq i} E_{ix}^j W_{jx}\right] + \frac{1}{4} W_{ji} \tag{26}$$

Finally, by symmetry, $E_{ji}^{j+1} = E_{ij}^{j+1}$. Noting that E_{ij}^k is undefined (and unnecessary) for $k < \max(i,j) + 1$, as nodes i and j have not both been added to the tree, (24) and (26) hence form a closed system. This can be solved inductively, finding all E_{ij}^m terms for $m = 2, 3, ..., n$.

Discrete minima.

Lemma 5: Define $f(\boldsymbol{v})$ to be the objective function for the discrete tree given by the Phylo2Vec vector $\boldsymbol{v}$. Then, if V^* is the set of vectors $\boldsymbol{v}$ which minimize f, any optimal W satisfies

$$\mathbb{P}(\boldsymbol{V} \in V^* | W) = 1 \tag{27}$$

Moreover if $|V^*| = 1$ then there is a unique $\boldsymbol{v}$ minimizing f, then there is a unique W minimizing F, which is the matrix such that

Proof: Define V to be the set of ordered tree vectors. Then,

$$F(W) = \sum_{\boldsymbol{u} \in V} \mathbb{P}(\boldsymbol{V} = \boldsymbol{u}|W) f(\boldsymbol{u}) = \sum_{\boldsymbol{u} \in V} \prod_{m=1}^{n-1} W_{m,u_m} f(\boldsymbol{u}) \quad \textbf{(28)}$$

As

$$\sum_{\boldsymbol{u} \in V} \mathbb{P}(\boldsymbol{V} = \boldsymbol{u}|W) = 1 \quad \textbf{(29)}$$

we see that $F(W)$ is a weighted average of the values of $f(\boldsymbol{u})$ for $\boldsymbol{u} \in V$. Thus, for any $\boldsymbol{v} \in V^*$

$$F(W) \geq f(\boldsymbol{v}) \quad \textbf{(30)}$$

and

$$F(W) = f(\boldsymbol{v}) \quad \Rightarrow \quad \mathbb{P}(\boldsymbol{V} \in V^*|W) = 1 \quad \textbf{(31)}$$

as required. If $V = \{\boldsymbol{v}\}$, this minimum requires

$$\mathbb{P}(\boldsymbol{V} = \boldsymbol{v}|W) = 1 \quad \textbf{(32)}$$

and therefore, using (28) W is uniquely defined by

$$W_{m,j} = \mathbb{I}\{v_m = j\} \quad \forall m, j \quad \textbf{(33)}$$

as required.

D. Orderings

This section considers the labelling algorithm introduced in the main text.

Lemma 6: Two nodes have the same label only if they share an ancestor with that label.

Proof: Suppose that this is false, and that nodes x and y have the same label, L, but do not share an ancestor with that label. Define $a(x)$ and $a(y)$ to be the nodes of lowest generation (that is, the nodes closest to the root) with label L such that they are ancestors of x and y respectively. Note that, by assumption, $a(x) \neq a(y)$ and also, neither can be the root (as the root is the ancestor of all nodes). Moreover, they cannot share a parent, as the children of a parent are labelled differently. Thus, without loss of generality, one can assume that $a(x)$ was labelled first. By definition, the parent of $a(x)$ does not have label L and hence, L must have been the smallest unused label when node $a(x)$ was labelled. A similar

argument for $a(y)$ shows that L must have been the smallest unused label when node $p(y)$ was labelled. However, when node $a(y)$ was labelled, L had been used to label $a(x)$, giving the required contradiction.

Lemma 7: l is a permutation of the set $\{0, 1, ..., n-1\}$.

Proof: Firstly, note that as there are $n-1$ internal nodes, and a single new label is introduced every time the children of an internal node are labelled, the set of labels used (across all nodes) must be $\{0, 1, ..., n-1\}$.

Suppose that leaf nodes x and y have the same label L. By Lemma 6, they must share an ancestor a with that label. Define b to be the shared ancestor with the highest generation (that is, furthest from the root) such that b has label L. Then, either nodes x and y are the children of b (in which case, they have distinct labels) or they are descendants of distinct children, c and d, of b. In the second case, one can impose without loss of generality that c does not have label L and, as label L has already been used to label b, we know that all descendants of c do not have label L. Hence, in both cases, one of x and y does not have label L as required.

Lemma 8: For each $i \in \{0, 1, ..., n-1\}$, define $x(i)$ to be the node of the highest generation with label i. Define (for $i > 0$), $y(i)$ to be the label of the parent of $x(i)$. Then, with ordering l, the tree is given by

$$v_0 = 0 \quad \text{and} \quad v_i = y(i) \quad \forall i > 0 \tag{34}$$

Proof: Firstly, note that $\boldsymbol{v}$ is ordered, as the label of a child is greater than or equal to that of its parent. Hence, as the parent of $x(i)$ does not have label i, it must have a label strictly less than i and so $v_i < i$ as required.

Consider constructing the tree according the "standard" right-to-left Phylo2Vec algorithm. One can then proceed by induction on the number of nodes. The case $n = 2$ is trivial, and so suppose it holds for $n = m$ and consider a tree with $n = m + 1$.

The first node to be processed has label m. No other node can have label m cannot also have label m (as, otherwise, one of its children would have label greater than m, contradicting Lemma 7) and hence, the first node to be processed is $x(m)$. The parent of $x(m)$ must have label $y(m)$. This must also be the label of its other child and hence, $v_m = y(m)$ ensures that the node with label m merges with its sibling from the original tree (which is correct).

From this point, the tree now has m nodes, and our right-to-left construction algorithm considers the parent of the node labelled m to now be a leaf node with label $y(m)$. The values of $y(i)$ for this tree are unchanged (in particular, $y(y(m))$ depends on the label of an ancestor of the parent of node m) and hence, by induction, the remaining values of $\boldsymbol{v}$ correctly generate the rest of the tree. Thus, the correct tree is generated by $\boldsymbol{v}$ as required.

E. Queue Shuffle: generating principled ordering proposals

Asymmetry of ordered tree spaces.

Lemma 9: Define g^k_m to be the expected distance from the root of the node labelled m in a tree with k nodes. Define the harmonic sum function

$$H(m) = \begin{cases} \sum_{j=1}^{m} \frac{1}{j} & \text{if } m \geq 1 \\ 1 & \text{if } m = 0 \end{cases} \tag{35}$$

Then,

$$g^k_m = H(k-1) + H(m) - 1 \tag{36}$$

Proof: From our left-to-right construction algorithm,

$$g^k_{k-1} = \sum_{m=0}^{k-2} \frac{1}{k-1}(1 + g^{k-1}_m) \tag{37}$$

as adding node $k-1$ according to $v_{k-1} = m$ means that the path length between node $k-1$ and the root will be one more than the path length of between m and the root. If $v_{k-1} = m$, it will also increase the path length between node m and the root by 1 and so

$$g^k_m = \frac{k-2}{k-1} g^{k-1}_m + \frac{1}{k-1}(g^{k-1}_m + 1) = g^{k-1}_m + \frac{1}{k-1} \tag{38}$$

The initial conditions of this system are that

$$g^2_0 = g^2_1 = 1 \tag{39}$$

as in a two-node rooted tree, the leaves are distance 1 from the root.

We claim by induction on k that the solution to this system is (36).

Note that this holds for $k = 2$ as $H(1) = H(0) = 1$. Moreover, under the inductive hypothesis that it holds for a tree with $k-1$ nodes, for $m < k-1$,

$$g^k_m = g^{k-1}_m + \frac{1}{k-1} = H(k-2) + H(m) - 1 + \frac{1}{k-1} = H(k-1) + H(m) - 1 \tag{40}$$

and

$$g^k_{k-1} = \sum_{m=0}^{k-2} \frac{1}{k-1}(1 + g^{k-1}_m) \quad \textbf{(41)}$$

$$= \sum_{m=0}^{k-2} \frac{1}{k-1}(1 + H(k-2) + H(m) - 1) \quad \textbf{(42)}$$

$$= \sum_{m=0}^{k-2} \frac{1}{k-1}(H(k-2) + H(m)) \quad \textbf{(43)}$$

$$= H(k-2) + \frac{1}{k-1}\sum_{m=0}^{k-2} H(m) \quad \textbf{(44)}$$

$$= H(k-1) + \frac{1}{k-1}\sum_{m=1}^{k-2}\sum_{j=1}^{m} \frac{1}{j} \quad \textbf{(45)}$$

Now,

$$\sum_{m=1}^{k-2}\sum_{j=1}^{m} \frac{1}{m} = \sum_{j=1}^{k-2}\sum_{m=j}^{k-2} \frac{1}{j} = \sum_{j=1}^{k-2} \frac{k-1-j}{j} = (k-1)H(k-2) - (k-2) \quad \textbf{(46)}$$

and hence

$$g^k_{k-1} = H(k-1) + H(k-2) - \frac{(k-2)}{(k-1)} = H(k-1) + H(k-1) - 1 \quad \textbf{(47)}$$

as required.

Nearest Neighbour Interchange.

Definition 4: Define $\tau(\sigma)$ to be the space of possible trees given an ordering σ.

Definition 5: For a tree $\mathcal{T}$, define $Q(\mathcal{T})$ to be the random ordering generated by Queue Shuffle.

Lemma 10: Consider a tree $\mathcal{T}$ and suppose that another tree, $\mathcal{T}'$ is one NNI move away from $\mathcal{T}'$. Then

$$\mathbb{P}\Big[\mathcal{T}' \in \tau\Big(Q(\mathcal{T})\Big)\Big] \geq \frac{1}{4} \quad \textbf{(48)}$$

To facilitate the proof, we first note that for any permutation σ, by Lemma 13, that

$$\mathbb{P}\Big[Q(\mathcal{T}) = \sigma\Big] \in \Big\{0, \frac{1}{2^{n-1}}\Big\} \quad \textbf{(49)}$$

Now, we consider labelling the tree as shown in the left panel of Fig. S1. Suppose that the edge to which the four subtrees are joined has nodes i and j, with node i being a higher generation than node j. Suppose that the subtrees rooted at the child of j are labelled as $\mathcal{S}_j$ and $\mathcal{S}_c$. Suppose that the other child of i (that is, the child not equal to j) is the root of a subtree $\mathcal{S}_i$. Finally, suppose that the fourth subtree is labelled $\mathcal{S}_r$ (this is the subtree containing the root). A single NNI move in this context involves swapping a pair of subtrees from the set $\{\mathcal{S}_i, \mathcal{S}_j, \mathcal{S}_r, \mathcal{S}_c\}$.

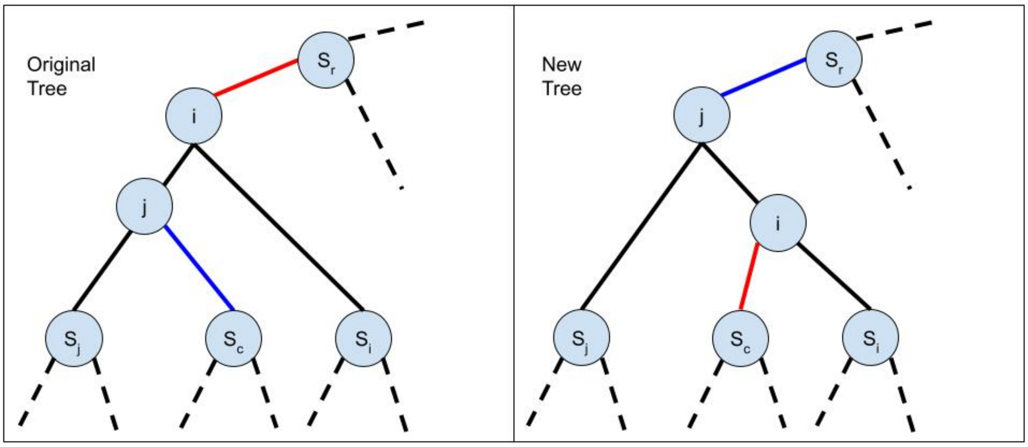

Figure S1. A simple representation of the swapping property of Queue Shuffle. The swapped subtrees have roots labelled S_i and S_j while the node label c is the subject of the equivalent subtree-prune and regraft operation. Note that in step 4, the internal nodes are renamed to illustrate that the required swap has indeed occurred.

By symmetry, swapping the pair $\mathcal{S}_j$ and $\mathcal{S}_c$ or the pair $\mathcal{S}_r$ and $\mathcal{S}_i$ has no impact, as the new tree will be topologically equivalent to $\mathcal{T}$ (which is always in the new shuffled space). Thus, one needs only to prove the result for the tree formed by swapping $\mathcal{S}_c$ and $\mathcal{S}_r$. This is topologically equivalent to the tree in the right panel of Fig. S1. We assume that this tree is $\mathcal{T}'$

For a given permutation σ, define $l_i(\sigma)$ to be the label assigned to node i, $l_j(\sigma)$ to be the label assigned to node j, $l_i^{\mathcal{S}}(\sigma)$ to be the label assigned to the root of $\mathcal{S}_i$, $l_j^{\mathcal{S}}(\sigma)$ to be the label assigned to the root of $\mathcal{S}_j$ and $l_c^{\mathcal{S}}(\sigma)$ to be the label assigned to the root of $\mathcal{S}_c$

Then, by Lemma 14,

$$\mathbb{P}\Big[l_j^{\mathcal{S}}(Q(\mathcal{T})) = l_i(Q(\mathcal{T}))\Big] = \frac{1}{4} \tag{50}$$

Now, suppose for a permutation σ that $l_j^{\mathcal{S}}(\sigma) = l_i(\sigma)$. Necessarily (as $r_B = 0$), $l_c^{\mathcal{S}}(\sigma)$ was the smallest available label when processing node j, which must be bigger than the smallest available label when node i was processed (as j is the child of i and hence processed later). Thus, $l_c^{\mathcal{S}}(\sigma) > l_i^{\mathcal{S}}(\sigma)$.

A further important result proved in Lemma 14 is that node j must have been placed ahead of the root of $\mathcal{S}_i$ in the queue. Thus, all nodes in $\mathcal{S}_i$ are labelled either as $l_i^{\mathcal{S}}(\sigma)$ or with a label that is greater than $l_c^{\mathcal{S}}(\sigma)$, as all internal nodes in $\mathcal{S}_i$ are processed after node j. Moreover, all nodes in $\mathcal{S}_j$ are labelled as either $l_j^{\mathcal{S}}(\sigma)$ or a label greater than $l_c(\sigma)$ as they were processed after node j.

Define $\boldsymbol{v}$ to be the vector giving $\mathcal{T}$ under σ. Then, define a new vector $\boldsymbol{v}'$ by

$$v'_m = \begin{cases} l_i^{\mathcal{S}}(\sigma) & \text{if } m = l_c^{\mathcal{S}}(\sigma) \\ v_m & \text{otherwise} \end{cases} \tag{51}$$

Then, $\boldsymbol{v}'$ is ordered as $l_c^{\mathcal{S}}(\sigma) > l_j^{\mathcal{S}}(\sigma)$. We now show that $\boldsymbol{v}'$ generates $\mathcal{T}'$ by using the left-to-right construction algorithm. Note that up to the point that the node labelled $l_c^{\mathcal{S}}(\sigma)$ is processed by this algorithm, no nodes have appended to the edges connecting either the root of $\mathcal{S}_i$ or $\mathcal{S}_j$ to the tree. This holds because all other nodes in $\mathcal{S}_i$ and $\mathcal{S}_j$ have labels greater than $l_c^{\mathcal{S}}(\sigma)$. Changing from $\boldsymbol{v}$ to $\boldsymbol{v}'$ means that the leaf node labelled $l_c^{\mathcal{S}}(\sigma)$ is joined to the edge connecting the root of $\mathcal{S}_i$ rather than the root of $\mathcal{S}_j$. The rest of the tree is then constructed identically as the vectors $\boldsymbol{v}$ and $\boldsymbol{v}'$ are the same.

Thus, changing from $\boldsymbol{v}$ to $\boldsymbol{v}'$ generates the tree in the right panel of Fig. S1, and therefore the tree formed by the NNI move swapping the trees $\mathcal{S}_c$ and $\mathcal{S}_r$.

This completes the proof as it shows that

$$\mathbb{P}\left[\mathcal{T}' \in \tau\Big(Q(\mathcal{T})\Big)\Big| l_j^{\mathcal{S}}(\sigma) = l_i(\sigma)\right] = 1 \tag{52}$$

and hence, by Lemma 14

$$\mathbb{P}\left[\mathcal{T}' \in \tau\Big(Q(\mathcal{T})\Big)\right] \geq \mathbb{P}\left[\mathcal{T}' \in \tau\Big(Q(\mathcal{T})\Big)\Big| l_j^{\mathcal{S}}(\sigma) = l_i(\sigma)\right]\mathbb{P}\left[l_j^{\mathcal{S}}(\sigma) = l_i(\sigma)\right] = \frac{1}{4} \tag{53}$$

F. Eutherian Mammal phylogeny (Song et al. 2012)

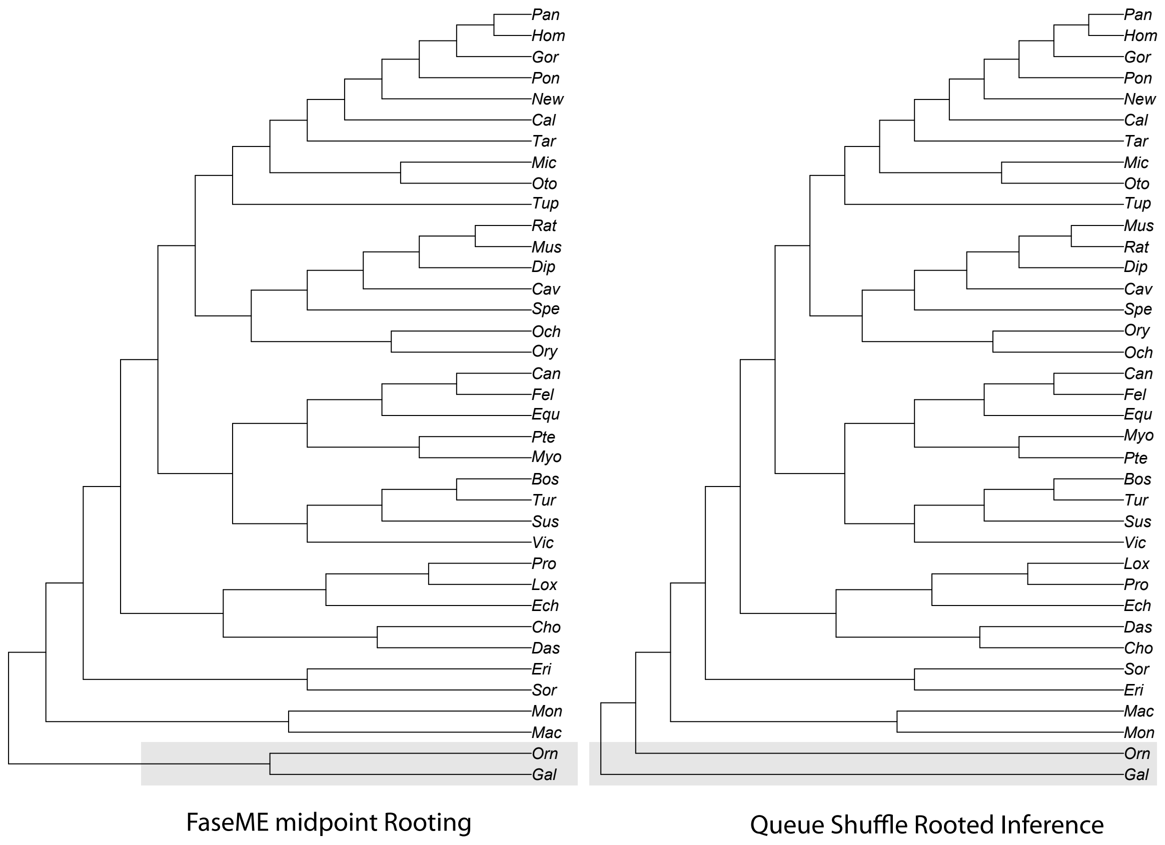

Figure S2. Comparison of the best unrooted FastME tree that has been midpoint rooted to an optimised rooted tree via Queue Shuffle on an Eutherian mammal dataset Song et al. 2012. Queue Shuffle correctly places Gallus gallus as the outgroup of mammals. Branch lengths are ignored and trees are displayed as ultrametric.

G. Convergence analysis

Figure S3 compares the performance of different optimisers (Adafactor (Shazeer and Stern 2018), AdamW (Loshchilov and Hutter 2019), RMSprop (Tieleman and Hinton 2012), and SGD) under different DNA substitutions models (JC69 (Jukes, Cantor, et al. 1969), F81 (Felsenstein 1981), TN93 (Tamura and Masatoshi Nei 1993)) on a single optimisation step (no subsequent reordering with Queue Shuffle) for a maximum of 5000 steps. Four learning rates were considered, logarithmically spaced from 0.001 to 1.0. Whereas convergence speed appears to be independent of the chosen DNA substitution model, the results varied widely with respect to the optimisation algorithm. In particular, Adafactor optimisation produced the best performance, with increasing convergence speed as learning rates were higher.

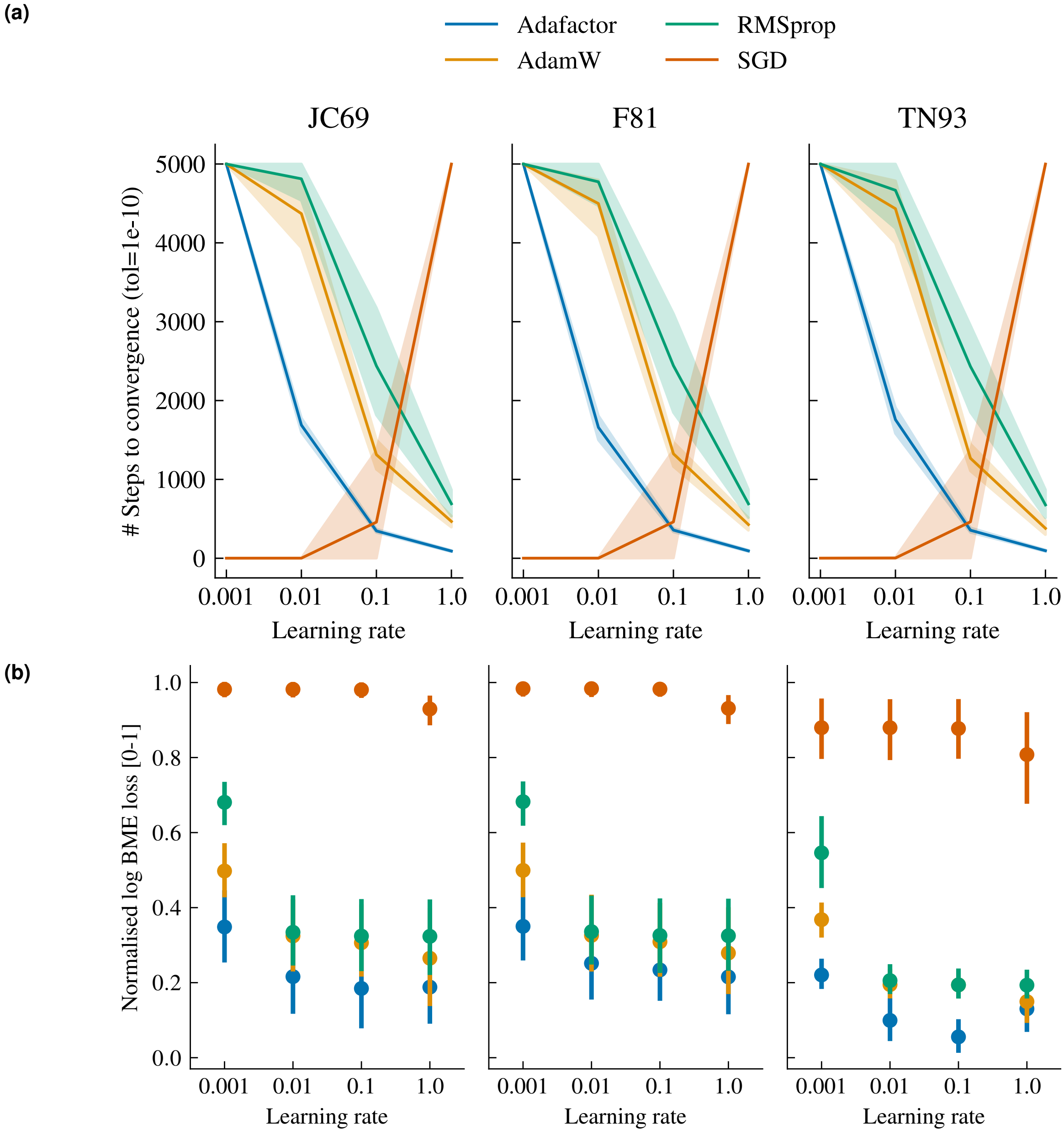

Figure S3. Convergence analysis of different optimisers and DNA substitution models for the datasets DS1-DS11 (see Table 2). **(a)** Number of steps needed to reach convergence (tolerance: 1e-10). **(b)** Loss reached at convergence. For each dataset, the log BME losses were scaled using min-max normalisation. Error bars denote 95% confidence intervals computed with 1000 bootstraps.

H. Miscallaneous Lemmas

Supplementary lemmas for Lemma 2.

Lemma 11: Using the notation of Lemma 2, define D_{Ar} to be the distance between node A and the root, and g_A to be the path length (i.e. the generation of node A). Then,

$$\sum_{X_b(A,B)=1} D_{AB} 2^{-e_{AB}} = 2 \sum_A 2^{-g_A} D_{Ar} \tag{54}$$

where the e_{AB} terms refer to the path lengths in the original unrooted tree.

Proof: We proceed by induction on the number of leaf nodes, n. For $n = 2$, the root must be placed on the edge connecting nodes 0 and 1 and $e_{01} = 1$. Then

$$\sum_{X_b(A,B)=1} D_{AB} 2^{-e_{AB}} = \frac{1}{2}(D_{01} + D_{10}) = \left(2^{1-1} D_{0r} + 2^{1-1} D_{1r}\right) \tag{55}$$

as required, noting that $D_{0r} + D_{1r} = D_{01}$. Suppose that our claim holds for $n = k$ and consider a tree with $n = k + 1$. Choose a pair of sibling leaf nodes (i.e. leaf nodes joined to the same internal node) y and z such that the root is not on an edge joining one of these nodes to the tree (this must exist as, for $n > 2$, there are at least two pairs of sibling leaf nodes). Suppose that y and z are connected to the internal node w. Now, note that the contribution to the objective of node y is

$$2 \sum_{B:X_b(y,B)=1} D_{yB} 2^{-e_{yB}} = 2 \sum_{B:X_b(w,B)=1} (D_{wB} + D_{wy}) 2^{-e_{yB}} \tag{56}$$

$$= 2 \sum_{B:X_b(w,B)=1} D_{wB} 2^{-e_{yB}} + 2D_{wy} \sum_{B:X_b(w,B)=1} 2^{-e_{yB}} \tag{57}$$

$$= \sum_{B:X_b(w,B)=1} D_{wB} 2^{-e_{wB}} + 2D_{wy} \sum_{B:X_b(w,B)=1} 2^{-e_{yB}} \tag{58}$$

where the factor of 2 comes from the fact that both (y, B) and (B, y) must be considered. The final term in this equation can be simplified. Consider the subtree whose leaves are the root and all nodes such that $X_b(w, B) = 1$. Then, by the Kraft Equality on this subtree

$$\sum_{B:X_b(w,B)=1} 2^{-g_B} = \frac{1}{2} \tag{59}$$

as g_B gives the distance between the root and node B. Moreover, as $e_{yB} = g_y + g_B - 1$ (where the -1 accounts for the fact that $\mathcal{T}$ is unrooted)

$$\sum_{B:X_b(w,B)=1} 2^{-e_{yB}} = 2^{1-g_y} \sum_{B:X_b(w,B)=1} 2^{-g_B} = 2^{-g_y} \tag{60}$$

Thus, (58) becomes

$$2\sum_{X_b(y,B)=1} D_{yB}2^{-e_{yB}} = \sum_{B:X_b(w,B)=1} D_{wB}2^{-e_{wB}} + 2D_{wy}2^{-g_y} \tag{61}$$

Thus, the change to $\sum_{X_b(A,B)=1} D_{AB}2^{-e_{AB}}$ caused by adding nodes y and z from the tree is to subtract

$$2\sum_{X_b(w,B)=1} D_{wB}2^{-e_{wB}} \tag{62}$$

(as node w is no longer a leaf node) and to add the contributions from nodes y and z

$$2\left(\sum_{B:X_b(y,B)=1} D_{yB}2^{-e_{yB}} + \sum_{B:X_b(z,B)=1} D_{zB}2^{-e_{zB}}\right) = \sum_{X_b(w,B)=1} D_{wB}2^{-e_{wB}} + 2D_{wy}2^{-g_y} + \sum_{X_b(w,B)=1} D_{wB}2^{-e_{wB}} + 2D_{wz}2^{-g_z} \tag{63}$$

There is no (y,z) term to consider as $X_b(y,z)=0$. Note that the $\sum_{X_b(w,B)=1} D_{wB}2^{-e_{wB}}$ terms cancel. Define $\mathcal{T}'$ to be the tree when node y and z are removed so, as it now has k leaf nodes, the inductive hypothesis can be used to show

$$\sum_{A,B\in\mathcal{T}:X_b(A,B)=1} D_{AB}2^{-e_{AB}} = \sum_{A',B'\in\mathcal{T}':X_b'(A',B')=1} D_{A'B'}2^{-e'_{A'B'}} + 2D_{wy}2^{-g_y} + 2D_{wz}2^{-g_z} \tag{64}$$

$$= 2\sum_{A'\in\mathcal{T}'} 2^{-g'_{A'}} D_{A'r'} + 2D_{wy}2^{-g_y} + 2D_{wz}2^{-g_z} \tag{65}$$

$$= 2\sum_{A'\in\mathcal{T}'/\{w\}} 2^{-g'_{A'}} D_{A'r'} + 2D_{wr}2^{-g_w} + 2D_{wy}2^{-g_y} + 2D_{wz}2^{-e_{zr}} \tag{66}$$

where dashes are used to denote quantities in $\mathcal{T}'$. Note that

$$\sum_{A'\in\mathcal{T}'/\{w\}} 2^{-g'_{A'}} D_{A'r'} = \sum_{A\in\mathcal{T}/\{y,z\}} 2^{-g_A} D_{Ar} \tag{67}$$

as only the nodes w, y and z are affected by changing from $\mathcal{T}$ to $\mathcal{T}'$. Moreover, note that

$$2^{-g_w} = 2^{-g_y} + 2^{-g_z} \tag{68}$$

as $g_w = g_y - 1 = g_z - 1$, and, as the distances are additive,

$$D_{wy} + D_{wr} = D_{yr} \quad \text{and} \quad D_{wz} + D_{wr} = D_{zr} \tag{69}$$

Hence

$$2D_{wy}2^{-g_y} + 2D_{wz}2^{-g_z} + 2D_{wr}2^{-g_w} = 2D_{wy}2^{-g_y} + 2D_{wz}2^{-g_z} + 2D_{wr}(2^{-g_y} + 2^{-g_z}) \tag{70}$$

$$= 2(D_{wy} + D_{wr})2^{-g_y} + 2(D_{wz} + D_{wr})2^{-g_z} \tag{71}$$

$$= 2D_{yr}2^{-g_y} + 2D_{zr}2^{-g_z} \tag{72}$$

Thus,

$$\sum_{X_b(A,B)=1} D_{AB}2^{-e_{AB}} = \sum_A 2^{-g_A} D_{Ar} \tag{73}$$

and hence the claim holds by induction as required.

Lemma 12: Using the notation of Lemma 2,

$$\mathcal{D} = \sum_A 2^{-g_A} D_{Ar} \tag{74}$$

Proof: This can be proved through induction on the number of nodes. It clearly holds if there are only two nodes in the tree. Assume it holds whenever there are k nodes, and consider a tree with $k+1$ nodes. We process the first pair of leaf nodes, y and z, and assume that their associated distances to their parent, w are D_{wy} and D_{wz} and that the new tree created is $\mathcal{T}'$ with distances D'. Then, we have, by our inductive hypothesis

$$\begin{aligned}
\mathcal{D} &= \sum_{A' \in \mathcal{T}'} 2^{-g'_{A'}} D'_{A'r'} && (75)\\
&= \sum_{A' \in \mathcal{T}'/\{w\}} 2^{-g'_{A'}} D'_{A'r'} + 2^{-g'_w} D'_{wr'} && (76)\\
&= \sum_{A \in \mathcal{T}/\{y,z\}} 2^{-g_A} D_{Ar} + 2^{-g_w}\left(D_{wr} + \frac{D_{wy}+D_{wz}}{2}\right) && (77)\\
&= \sum_{A \in \mathcal{T}/\{y,z\}} 2^{-g_A} D_{Ar} + 2^{-g_w}\left(\frac{D_{yr}+D_{zr}}{2}\right) && (78)\\
&= \sum_{A \in \mathcal{T}} 2^{-g_A} D_{Ar} && (79)
\end{aligned}$$

as required, where we have used the fact that $D_{wr} + D_{wy} = D_{yr}$ and that $2^{-g_w} = 2 \times 2^{-g_y} = 2 \times 2^{-g_z}$.

Supplementary Lemmas for Lemma 10.

Lemma 13: Using the notation of Lemma 10, for a given permutation σ,

$$\mathbb{P}\Big[Q(\mathcal{T}) = \sigma\Big] \in \left\{0, \frac{1}{2^{n-1}}\right\} \tag{80}$$

Proof Suppose that every (internal and external) node in $\mathcal{T}$ is assigned a fixed unique position, such that one can consistently define a "leftmost" and "rightmost" node from a pair of nodes. Then, the randomness in the Queue Shuffle algorithm can be represented by a uniform random vector $\boldsymbol{r} \in \{0,1\}^{n-1}$ such that when the mth internal node is processed, the leftmost child is given the same label as its parent if and only if $r_{m+1} = 1$. Each distinct $\boldsymbol{r}$ results in a distinct ordering (as, given the leaf labels, one can generate the unique labelling of the tree as a parent must have a label equal to the minimum of the labels of its two children). Hence, each possible labelling is equally likely, giving (80) and completing the proof.

Lemma 14: Using the notation of Lemma 10

$$\mathbb{P}\left[l_j^{\mathcal{S}}(Q(\mathcal{T})) = l_i(Q(\mathcal{T}))\right] = \frac{1}{4} \tag{81}$$

Moreover, $l_j^{\mathcal{S}}(Q(\mathcal{T})) = l_i(Q(\mathcal{T}))$ if and only if node j was placed ahead of the root of $\mathcal{S}_i$ in the queue

Proof Using the $\boldsymbol{r}$ defined in the proof of Lemma 13, define the random indices A and B such that r_A corresponds to processing node i and r_B corresponds to processing node j. Note that r_A and r_B are independent (though B will in general depend on r_A).

Now, suppose without loss of generality that j is the leftmost child of i and that the root of $\mathcal{S}_j$ is the leftmost child of j. As children have labels greater than or equal to their parents,

$$\mathbb{P}\left[l_j^{\mathcal{S}}(Q(\mathcal{T})) = l_i(Q(\mathcal{T}))\right] = \mathbb{P}\left[l_j^{\mathcal{S}}(Q(\mathcal{T})) = l_j(Q(\mathcal{T})) = l_i(Q(\mathcal{T}))\right] \tag{82}$$

and hence

$$\mathbb{P}\left[l_j^{\mathcal{S}}(Q(\mathcal{T})) = l_i(Q(\mathcal{T}))\right] = \mathbb{P}(r_A = r_B = 1) = \frac{1}{4} \tag{83}$$

which is the first result. The second result follows immediately from the fact that $r_A = 1$.

I. Estimation of GTR+Γ distances

To estimate distances under a GTR+Γ substitution model we use the approach outlined in (Yang 2006). Assuming a general time reversible rate symmetric matrix Q, the transition probability matrix over time is found via the matrix exponential $P(t) = e^{Qt}$. This matrix exponential can be readily computed via eigendecomposition.

Including variable rates among sites for a Gamma distribution g, $P(t) = \int e^{Qu} g(u)du$, which can again be estimated via eigendecomposition. Given parameters for rates, S, frequencies, π, the time between two sequences t_{ij}, and genetic sequence data $\mathcal{G}$, the log-likelihood for the transitions between a pair of taxa i and j is

$$\mathcal{L}_{ij}(\mathcal{G}|S, \pi, t_{ij}) = \sum_a \sum_b \kappa_{ab}^{ij} \log(P_{ab}(t_{ij}; S, \pi)) \tag{84}$$

where κ_{ab}^{ij} is the number of $a \to b$ transitions from taxon i to taxon j. We approximate the optimal parameters by maximizing the total log-likelihood (that is, the sum over i and j of $\mathcal{L}_{ij}$) using gradient descent in Jax.

J. Fast discrete hill-climbing with Phylo2Vec

The computational complexity of GradME is substantially higher than that of FastME due to the continuous nature of the algorithm. Thus, particularly for large numbers of taxa, using a similarly fast algorithm, at least to get close to the optimal tree, may be preferable.

Because of this, we have developed an alternative, discrete algorithm which has the same computational complexity as FastME. At each step, our algorithm outputs a matrix Δ such that Δ_{ij} is the change in the objective function if the value of v_i were changed to be equal to j. Δ allows us to perform a hill-climbing optimisation, as the change corresponding to the minimum value of Δ is made.

Analogously to FastME, Δ can be calculated in $\mathcal{O}(n^2 \text{diam}(\mathcal{T}))$ time, where $\text{diam}(\mathcal{T})$ is the maximum inter-taxa path length of the tree (this is generally substantially smaller than n). This algorithm works exclusively for unrooted trees, though a similar algorithm could be developed using our rooted objective.

Motivated by the method of (Desper and Gascuel 2002), our algorithm begins by calculating directed edge weight vectors $\boldsymbol{w}_e^{\pm}$ for the edges in $\mathcal{T}$. Each edge e naturally partitions $\mathcal{T}$ into two disjoint subtrees, $\mathcal{S}_1^e$ and $\mathcal{S}_2^e$, with each one being rooted at a node connected to e. We suppose that $\mathcal{S}_1^e$ is the tree not containing some fixed node X. Then, we assign $\boldsymbol{w}_e^{+}$ to be the balanced distance between the root of $\mathcal{S}_1^e$ and each of the individual leaf nodes in $\mathcal{S}_2^e$, and $\boldsymbol{w}_e^{-}$ to be the balanced distance between the root of $\mathcal{S}_2^e$ and each of the individual leaf nodes in $\mathcal{S}_1^e$. These edge weights can be calculated efficiently by using an iterative scheme, using the fact that the weight on a given edge from an internal node x to another node y is the mean of the weights of the two edges from nodes z and a (both distinct from y) to x (recalling that these weights are directional). This iterative scheme is closed by the fact that the weights on edges from leaf nodes to the tree are given by the appropriate columns of the distance matrix D.

This weighted tree can be used to calculate the distance between any pair of subtrees. For each fixed subtree, $\mathcal{S}$, one can iteratively calculate the distance $d_{\mathcal{S}\mathcal{R}}$ between it and (disjoint) subtrees $\mathcal{R}$, using the fact that the precalculated $\boldsymbol{w}$ terms give the distance between each subtree and each leaf, and that for any pair of subtrees $\mathcal{R}_1$ and $\mathcal{R}_2$ which share a parent node and can therefore be combined in a subtree $\mathcal{R}_3$,

$$d_{\mathcal{S}\mathcal{R}_3} = \frac{1}{2}\left(d_{\mathcal{S}\mathcal{R}_1} + d_{\mathcal{S}\mathcal{R}_2}\right) \tag{85}$$

From (Desper and Gascuel 2002), there exists a simple formula for the difference in objective function caused by pruning a subtree and regrafting it to an adjacent edge. If an internal node x is joined to nodes which are the roots of disjoint subtrees A, B and C, then the difference between attaching a subtree K to the edge joining x and C and attaching the subtree K to the edge joining x and B is

$$\frac{1}{4}\left(\delta_{AB} + \delta_{KC} - \delta_{AC} - \delta_{KB}\right) \tag{86}$$

where here, δ denotes inter-subtree distance. These δ terms are not equivalent to the d terms, as removing K from the original tree creates a different set of possible subtree pairs. However, they can be calculated in $\mathcal{O}(n^2 \text{diam}(\mathcal{T}))$ time from the distances d following the methods of (Desper and Gascuel 2002).

Explicitly, our algorithm moves outwards from the parent of the subtree which we are removing. We take B to be the node we are currently processing, x to be the previously processed node on this path, C to the node processed two iterations ago, and A to be the other node connected to x. Then, one has simply

$$\delta_{AB} = d_{AB} \quad \text{and} \quad \delta_{KB} = d_{KB} \tag{87}$$

Calculating δ_{AC} is slightly more complicated as the subtree K has been removed from C. If the original root of K was a path length l from C, and if that root shares a parent node (in C) with a subtree F, then

$$\delta_{AC} = d_{AC} + 2^{-l}(d_{AF} - d_{AK}) \tag{88}$$

as the subtree F now has double the weight in C. Finally, to calculate d_{CK}, one must calculate the distance between K and all subtrees connected to, but disjoint from the path taken from the tree root to C. If these subtrees are $\mathcal{S}_1, ..., \mathcal{S}_m$ respectively and are distance $1, ..., m$ from C, then

$$\delta_{KC} = \sum_{q=1}^{m} 2^{-q} d_{C\mathcal{S}_q} \tag{89}$$

It is this step which pushes the complexity of our algorithm above n^2

Thus, one can calculate the objective differences caused by each subtree-prune and regraft move by repeatedly applying this formula for each pruned subtree, essentially “moving” this subtree around the remaining tree.

The final step of our algorithm is to find the corresponding subtree-prune and regraft move for each possible change of $\boldsymbol{v}$. When v_i is changed to be equal to j, the subtree that is moved is the largest subtree of $\mathcal{T}$ containing i such that the leaves all have labels greater than or equal to i (in many cases, this will simply be the node i). For each value of i, one can find the edges which were connected to each leaf node when node i was attached in the left-to-right construction algorithm. The edge connected to node j at this stage gives the location of the regraft position of the subtree.

K. Code: continuous BME objective function

```python
import jax.numpy as np

from jax import jit, lax
from jax.scipy.special import logsumexp

@jit
def get_edges_exp_log(W, rooted):
    """Calculate the log-expectation of the objective value of a tree drawn with distribution W.
    We calculate and update E_ij throughout the left-to-right construction procedure

    Args:
        W (jax.numpy.array): Tree distribution
        rooted (bool): True is the tree is rooted, otherwise False

    Returns:
        E (jax.numpy.array): Log of the expected objective value of a tree drawn with W
    """
    # Add jnp.finfo(float).eps to W.tmp to avoid floating point errors with float32
    W_tmp = (
        jnp.pad(W, (0, 1), constant_values=jnp.finfo(float).eps) + jnp.finfo(float).eps
    )

    n_leaves = len(W) + 1

    E = jnp.zeros((n_leaves, n_leaves))
    E = E.at[1, 0].set(
        0.5 * E[0, 0] * W_tmp[0, 0] + jnp.log(0.25 * (2 - rooted) * W_tmp[0, 0])
    )
    E = E + E.T

    trindx_x, trindx_y = jnp.tril_indices(n_leaves - 1, -1)

    def body(carry, _):
        E, i = carry

        E_new = jnp.zeros((n_leaves, n_leaves))

        trindx_x_i = jnp.where(trindx_x < i, trindx_x, 1)
        trindx_y_i = jnp.where(trindx_x < i, trindx_y, 0)

        indx = (trindx_x_i, trindx_y_i)

        E_new = E_new.at[indx].set(
            E[indx] + jnp.log(
                1 + jnp.finfo(float).eps - 0.5 * (W_tmp[i - 1, indx[1]] + W_tmp[i - 1, indx[0]])
            )
        )

        # exp array
        mask_Ei = jnp.where(jnp.arange(n_leaves) >= i, 0, 1)
        exp_array = E * jnp.where(jnp.arange(n_leaves) >= i, 0, mask_Ei.T)

        # coef array
        mask_Wi = jnp.where(jnp.arange(n_leaves) >= i, 0, 0.5 * W_tmp[i - 1])
```

```
        coef_array = (jnp.zeros_like(W_tmp) + mask_Wi).at[:, i].set(0.25 * W_tmp[i - 1])
        coef_array = coef_array * (1 - jnp.eye(W_tmp.shape[0]))

        # logsumexp
        tmp = logsumexp(exp_array, b=coef_array, axis=-1) * mask_Ei

        E_new = E_new.at[i, :].set(tmp)

        # Update E
        E = E_new + E_new.T

        return (E, i + 1), None

    (E, _), _ = lax.scan(body, (E, 2), None, length=n_leaves - 2)

    return E

@jit
def bme_loss_log(W, D, rooted):
    """Log version of the BME loss function

    Args:
        W (jax.numpy.array): Tree distribution
        D (jax.numpy.array): Distance matrix
        rooted (bool): True is the tree is rooted, otherwise False

    Returns:
        loss (float): BME loss
    """
    E = get_edges_exp_log(W, rooted)
    loss = logsumexp(E, b=D)
    return loss
```
