# Supplementary figures and images for: Leaping through Tree Space: Continuous Phylogenetic Inference for Rooted and Unrooted Trees
Source: Genome Biol Evol. 2023 Dec 12;15(12):evad213. doi: 10.1093/gbe/evad213 (PMC10745275; doi:10.1093/gbe/evad213)

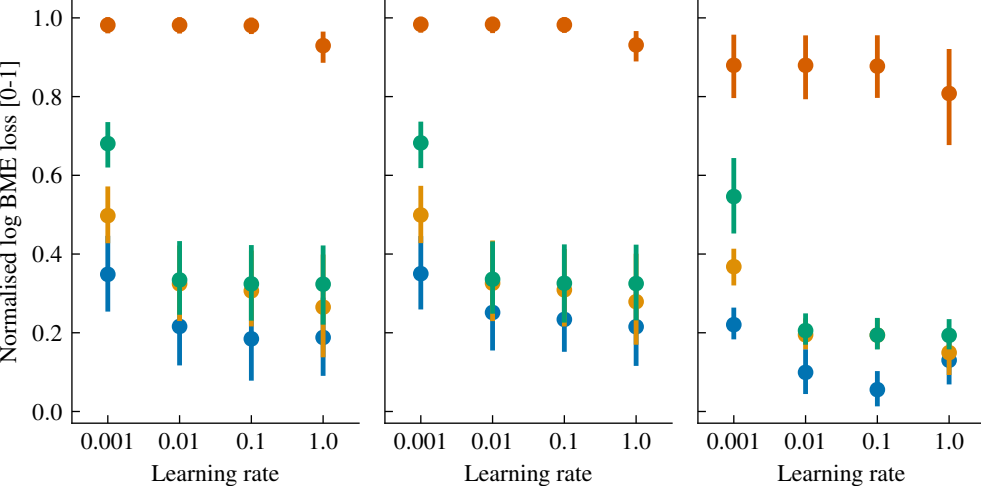

Supplement: evad213_Supplementary_Data [file evad213_supplementary_data.zip › FigS3b.pdf]

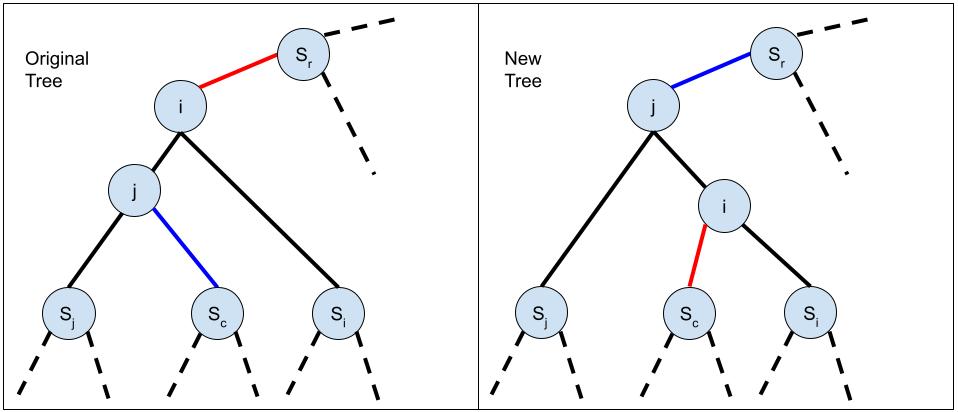

Supplement: evad213_Supplementary_Data [file evad213_supplementary_data.zip › FigS1.jpg]

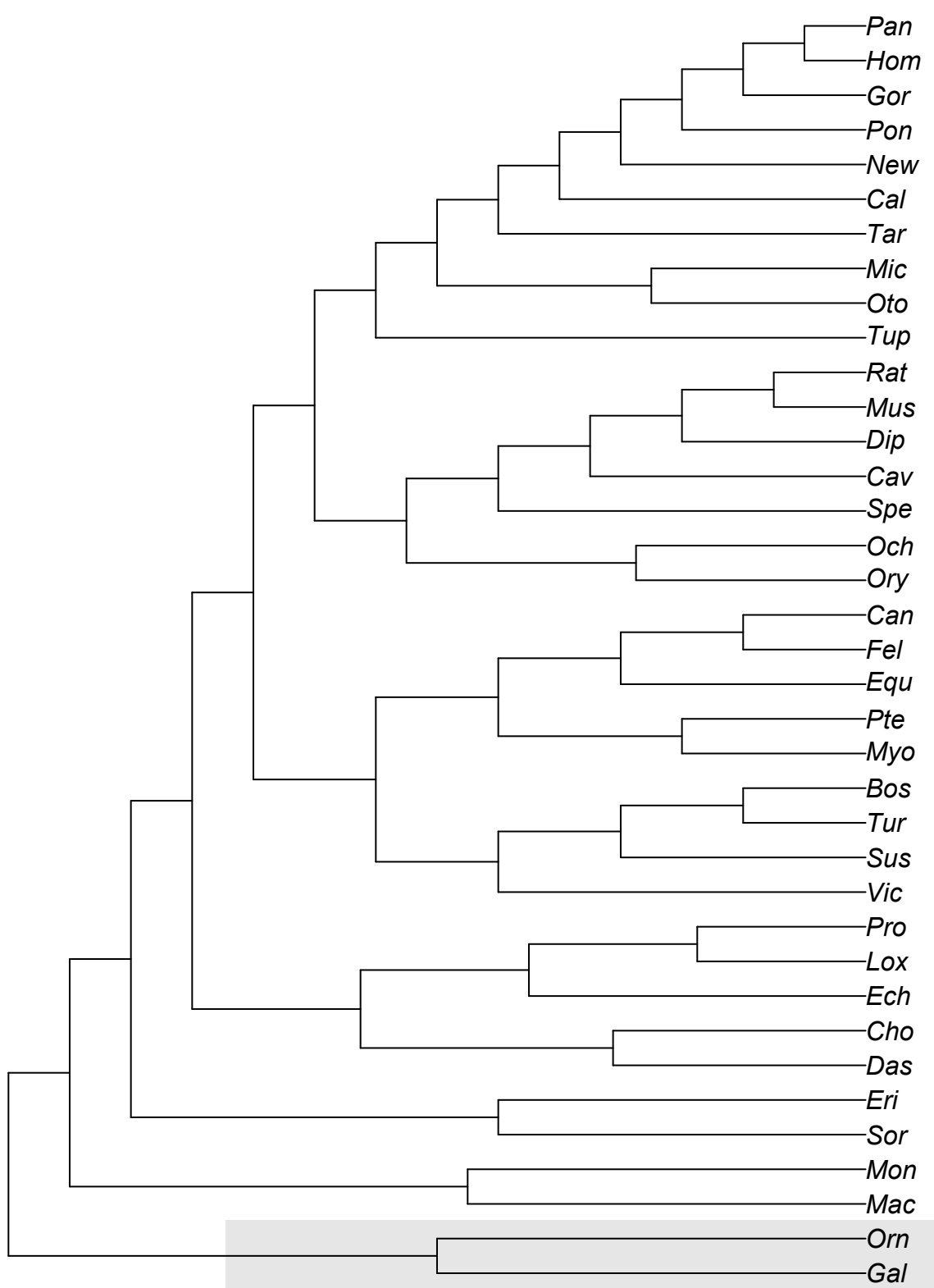

## FaseME midpoint Rooting

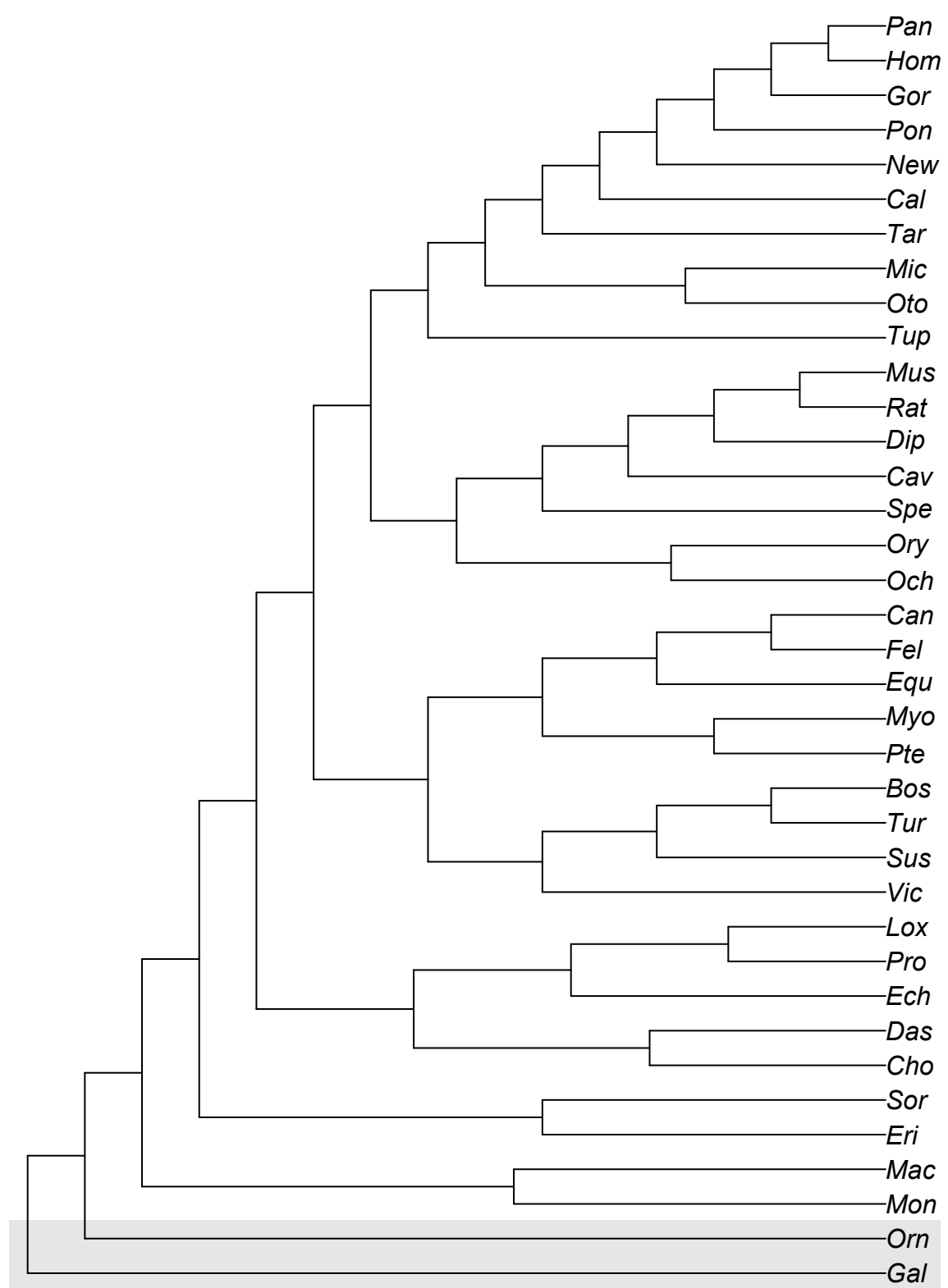

## Queue Shuffle Rooted Inference

Supplement: evad213_Supplementary_Data [file evad213_supplementary_data.zip › FigS2.pdf]

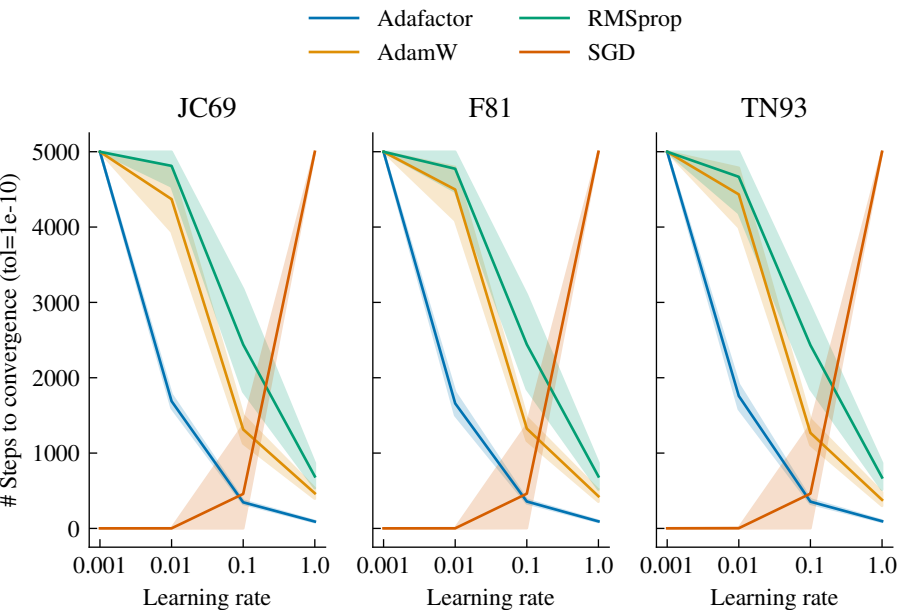

Supplement: evad213_Supplementary_Data [file evad213_supplementary_data.zip › FigS3a.pdf]
